# The importance of tyrosines in multimers of cyclic RGD nonapeptides: towards αvβ6-integrin targeted radiotherapeutics†

**DOI:** 10.1039/d4md00073k

**Published:** 2024-04-19

**Authors:** Neil Gerard Quigley, Maximilian Alexander Zierke, Beatrice Stefanie Ludwig, Frauke Richter, Nghia Trong Nguyen, Falco Reissig, Jakub Šimeček, Susanne Kossatz, Johannes Notni

**Affiliations:** a Institute of Pathology, School of Medicine and Health, Technische Universität München Munich Germany; b Department of Nuclear Medicine, University Hospital Klinikum Rechts der Isar and Central Institute for Translational Cancer Research, (TranslaTUM), School of Medicine and Health, Technische Universität München Munich Germany; c TRIMT GmbH Carl-Eschebach-Str. 7 D-01454 Radeberg Germany jn@trimt.de

## Abstract

In a recent paper in this journal (*RSC Med. Chem.*, 2023, **14**, 2429), we described an unusually strong impact of regiospecific exchange of phenylalanines by tyrosines in 10 gallium-68-labeled trimers of certain cyclic RGD peptides, c[XRGDLAXp(*N*Me)K] (X = F or Y), on non-specific organ uptakes. We found that there was, in part, no correlation of liver uptake with established polarity proxies, such as the octanol–water distribution coefficient (log *D*). Since this observation could not be explained straightforwardly, we suggested that the symmetry of the compounds had resulted in a synergistic interaction of certain components of the macromolecules. In the present work, we investigated whether a comparable effect also occurred for a series of 5 tetramers labeled with lutetium-177. We found that in contrast to the trimers, liver uptake of the tetramers was well correlated to their polarity, indicating that the unusual observations along the trimer series indeed was a unique feature, probably related to their particular symmetry. Since the Lu-177 labeled tetramers are also potential agents for treatment of a variety of αvβ6-integrin expressing cancers, these were evaluated in mice bearing human lung adenocarcinoma xenografts. Due to their tumor-specific uptake and retention in biodistribution and SPECT imaging experiments, these compounds are considered a step forward on the way to αvβ6-integrin-targeted anticancer agents. Furthermore, we noticed that the presence of tyrosines in general had a positive impact on the *in vivo* performance of our peptide multimers. In view of the fact that a corresponding rule was already proposed in the context of protein engineering, we argue in favor of considering peptide multimers as a special class of small or medium-sized proteins. In summary, we contend that the performance of peptide multimers is less determined by the *in vitro* characteristics (particularly, affinity and selectivity) of monomers, but rather by the peptides' suitability for the overall macromolecular design concept, and peptides containing tyrosines are preferred.

## Abstract 2

Tyrosines preferred: tetramers of αvβ6-integrin binding cyclopeptides show more favorable biodistribution (less nonspecific uptake) the more tyrosines they contain, which can not be deduced from the *in vitro* properties of the peptide monomers.

## Graphical abstract

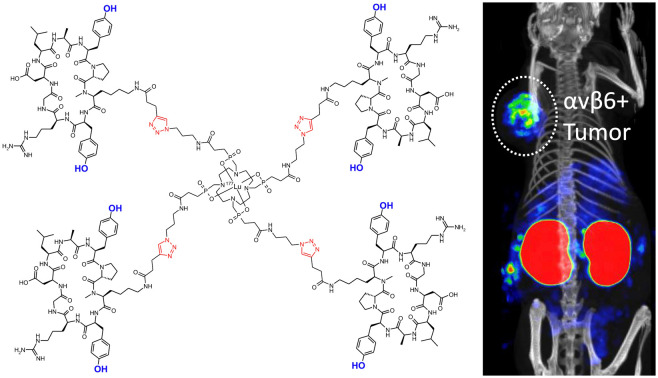

## Introduction

Peptide multimers have gained a firm place in the canon of compound classes used in radiopharmacy.^1^ This is because the combination of multiple copies of the same binding motif for a biological target in a single molecule increases avidity for that target—a principle that nature has always exploited in the form of regular antibodies, which are nothing else than dimers of targeting proteins.^2^ A particularly important component of a synthetic multimer is the scaffold, because its valency, geometry, and symmetry determine multiplicity, distance, and interaction between the conjugated functional monomers.^3,4^ The spatial distribution and motility of the comprised monomeric ligands can furthermore be adjusted using linkers of different lengths and rigidity to optimize avidity,^5,6^ the latter being considered a cumulative effect of individual target binding events.^7^ This strategy has been extensively exploited in connection with perhaps the most widely used class of ligands for αvβ3-integrin, the cyclic pentapeptides c(RGDxX) (x = f, y; X = K, E).^8^ Clinical data have suggested that radiolabeled c(RGDxK) dimers^9^ outperform the respective monomers for αvβ3-integrin targeted positron emission tomography (PET) diagnostics in patients.^10^ Comparative studies revealed that multimers with more than two c(RGDxK) units possess even higher avidities than monomers and dimers,^11–13^ which frequently also resulted in higher target-specific uptakes *in vivo*.^14–16^ This design principle has been applied to various targeting moieties with high relevance in radiopharmacy, such as fibroblast activation protein inhibitors (FAPi)^17^ or prostate-specific membrane antigen (PSMA) inhibitors,^18–20^ although it did not always lead to improved *in vivo* properties.^21^ However, the concept generally works well for radiopharmaceuticals based on peptides and peptidomimetics targeting integrins,^22^ such as αvβ3,^14–16^ α5β1,^23,24^ αvβ8,^25,26^ or αvβ6.^27,28^ The approach certainly is a valuable addition to the radiopharmaceutical toolbox,^1^ as it has been shown that even peptides whose affinities are too low for the radiolabeled monomers to be used as radiotracers can be useful for effective *in vivo* addressing when their target-specific binding is boosted by multimerization.^26,28,29^

However, multimerization does not only enhance avidity to the desired target but may also amplify unknown weak interactions.^30^ Peptide multimers therefore can exhibit altered target specificity or unexpectedly bind to sites not recognized by the respective monomers, which makes the successful design of multimeric probes and therapeutics a challenging endeavour. In addition, they differ from oligopeptides in that they have several times the molecular weight, which puts them on the borderline of proteins. Together with their sometimes altered polarity, they can therefore exhibit significant differences in pharmacokinetic parameters such as tissue penetration, elimination pathways and kinetics, and plasma half-life.^31^ Boehmer *et al.* have furthermore pointed out that multimers are characterized by a higher complexity that needs to be better understood to enable a rational design of clinically applicable agents.^32^ The increased complexity of multimers apparently leads to a higher degree of indeterminancy in the structure–activity relationships, because the components of complex systems can interact in unpredictable ways and thus produce completely new properties, which is known as emergence.^33^ Concerning multimers, this means that such emergent properties cannot in principle be calculated or otherwise derived straightforwardly from data for the respective monomers. Comprehensive data collection for libraries of multimers seems to be the only way to close this gap in understanding.^34^

One aspect of multimerization that is particularly difficult to predict is nonspecific accumulation in non-target organs and tissues, which can emerge unexpectedly and render an agent with otherwise promising properties, such as high avidity and target-specific uptake, virtually useless.^35^ Although most binding motifs are well characterized in terms of their specificity for their respective pharmacological target, usually only a small fraction of the multitude of possible interactions with other cell types, surface proteins, transporters, enzymes, or other biological contact sites in a living organism is known, which of course applies all the more the larger the respective molecule and thus the greater the number of possible contact sites.

**Fig. 1:**
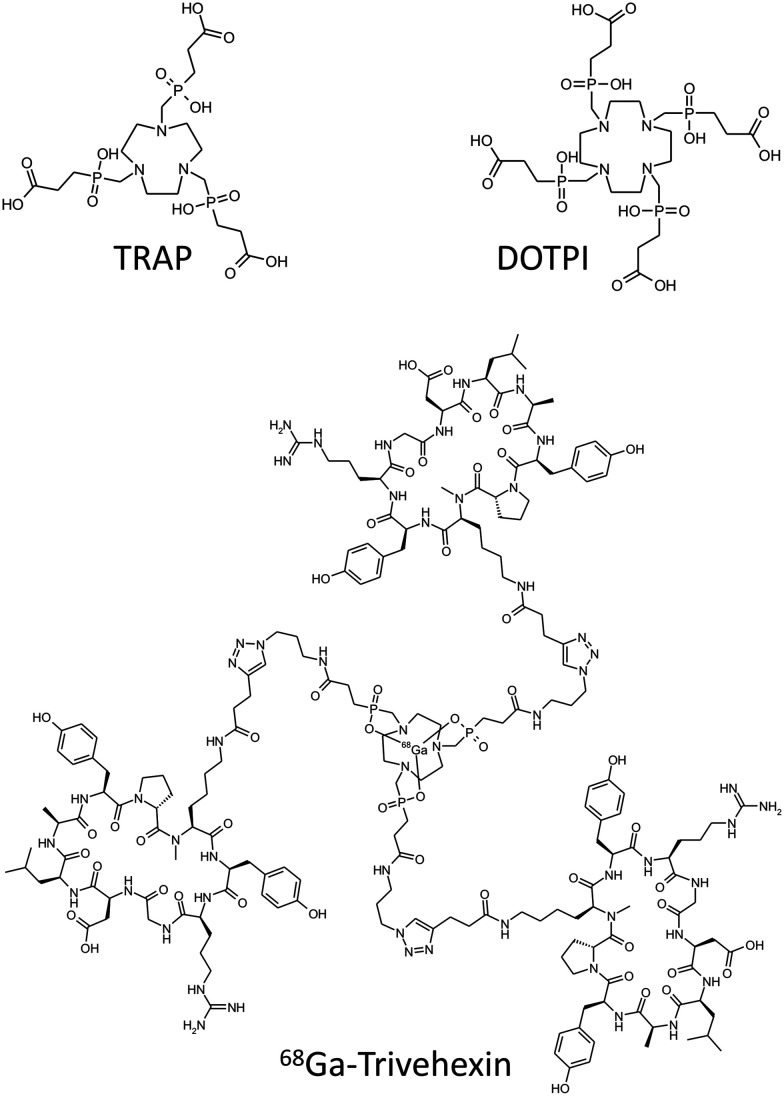
Structures of compounds discussed in the text.

To this end, we recently investigated the influence of small structural variations on the biodistribution of the αvβ6-integrin targeted positron emission tomography (PET) radiopharmaceutical ^68^Ga-Trivehexin (Fig. 1).^36^ This molecule comprises three copies of the cyclic nonapeptide c[YRGDLAYp(*N*Me)K], which are attached to a central triazacyclononane-triphosphinate (TRAP, see Fig. 1)^37^ structure that serves as both a highly efficient chelator for ^68^Ga^38^ and a C_3_-symmetrical trimeric scaffold.^39^^68^Ga-Trivehexin thus comprises 6 tyrosines, which we regioselectively and consecutively substituted by phenylalanines.^34^ The influence of these substitutions on the biodistribution proved to be counterintuitive to some extent. For example, although a high non-specific liver uptake of the all-Phe derivative was reduced by 50% upon introduction of a single Tyr, and by almost three quarters by two Tyr's, there was no concordant change in polarity proxies, such as log *D*. We concluded that non-specific tissue uptake is most likely an emergent phenomenon arising from synergistic interaction of phenyl groups in these highly symmetric molecules.^34^ One question that arose in the course of this study was whether the observed correlations are primarily caused by the highly ordered triangular symmetry of the compounds, or whether comparable effects can also be reproduced within a similar set of structures that exhibit a different symmetry.

In order to shed more light on this question, the present study takes a closer look at comparable structures based on the DOTPI framework (Fig. 1).^40^ This tetrameric scaffold is a larger congener of TRAP and can be functionalized in a similar manner, *i.e.*, by virtue of the copper(i)-catalyzed alkyne–azide cycloaddition (CuAAC) using the respective azide-functionalized derivative,^41,42^ which provides access to symmetrically and asymmetrically substituted peptide tetramers in practical amounts.^43^ DOTPI rapidly forms kinetically inert complexes with many radiometal ions that emit particle radiation, such as the alpha emitter ^213^Bi^III^,^44^ or trivalent radiolanthanides and especially the popular beta emitter ^177^Lu^III^.^40^ We herein report synthesis and preclinical data (*ex vivo* biodistribution and SPECT imaging in tumor-bearing mice) for tetrameric conjugates of αvβ6-integrin binding cyclic nonapeptides with the sequence cyclo[XRGDLAXp(*N*Me)K] (X = F or Y), which also constitutes the next step towards αvβ6-integrin targeted radiotherapeutics on the basis of these peptides. Such agents are currently considered of high clinical relevance^45,46^ because αvβ6-integrin is upregulated in various malignant cancers,^47^ especially in pancreatic ductal adenocarcinoma (PDAC),^48^ oral squamous cell carcinoma (OSCC),^49^ ovarian^50^ and cervical cancer,^51^ as well as in primaries and brain metastases^52^ of non-small cell lung cancer (NSCLC).^53^

## Results

### Peptide conjugation *via* CuAAC

**Fig. 2:**
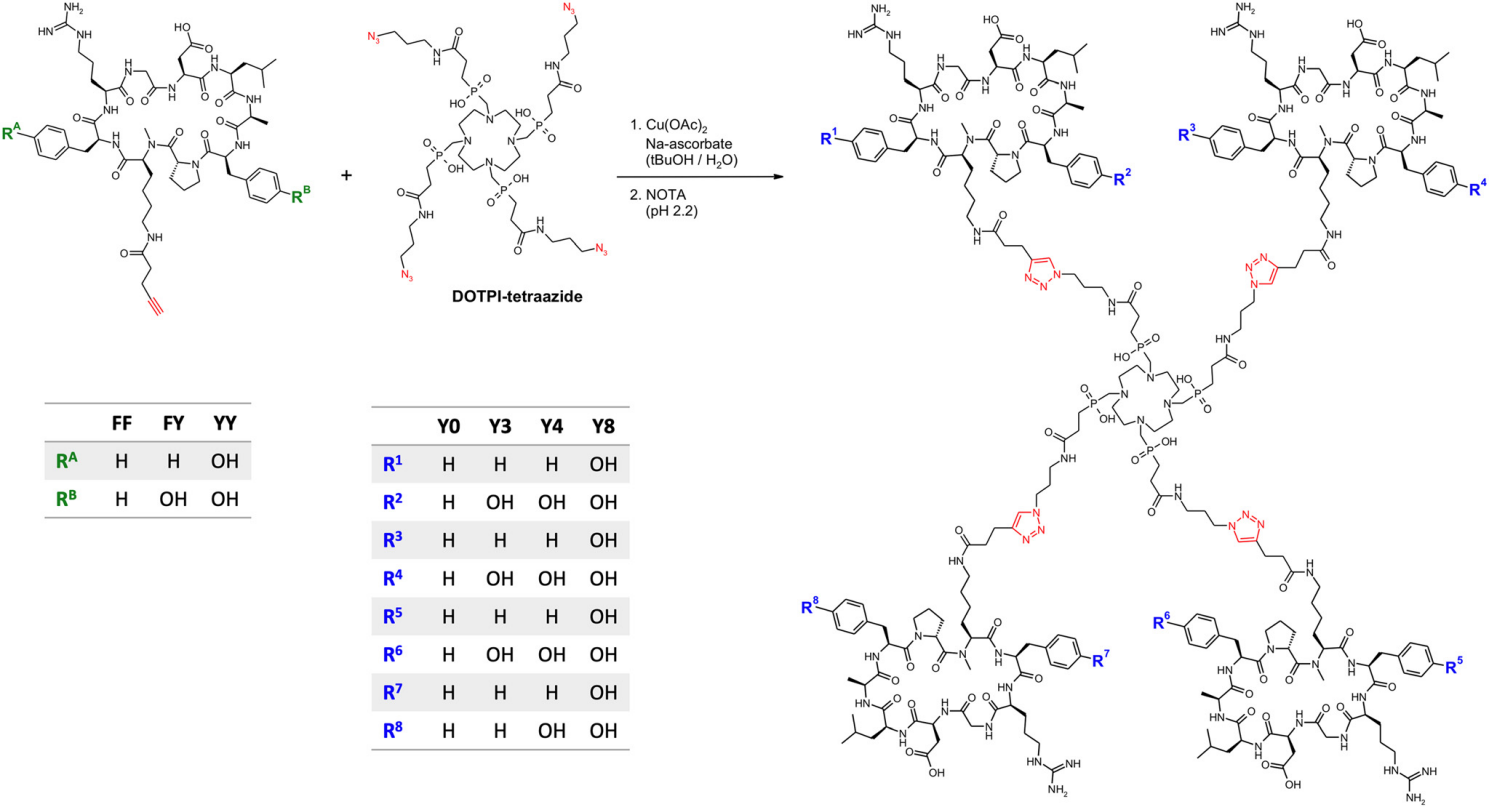
Synthesis of tetrameric conjugates of αvβ6-integrin binding peptides. Synthesis of **Y0**, **Y4**, and **Y8** was done by reacting DOTPI-tetraazide with the peptides **FF**, **YF**, and **YY**, respectively. The asymetrical conjugate **Y3** was isolated from the CuAAC reaction of DOTPI-tetraazide with a mixture of **FF** and **YF** by means of preparative HPLC. Functional groups related to the CuAAC are highlighted in red.

Monomeric peptide building blocks **FF**,^29^**FY**,^34^ and **YY**^36^ (Fig. 2) were synthesized as described previously by coupling 4-pentynoic acid to the Lys side chains of the three cyclopeptides cyclo[FR(Pbf)GD(*t*Bu)LAFp(*N*Me)K], cyclo[FR(Pbf)GD(*t*Bu)LAY(*t*Bu)p(*N*Me)K], or cyclo[Y(*t*Bu)R(Pbf)GD(*t*Bu)LAY(*t*Bu)p(*N*Me)K], followed by acidic deprotection. With regard to our preceding study on comparable trimeric peptides,^34^ we found that the increase in possible Phe → Tyr exchange positions and the change in symmetry leads to an even greater number of possible isomers, rendering a full-scale *in vivo* evaluation unfeasible. The imperative to reduce animal experiments to a reasonable minimum forced us to limit this investigation to the most relevant examples. We therefore refrained from including the fourth possible monomer **YF** (in which R^A^ is OH and R^B^ is H, see Fig. 2) into this investigation because in the preceding study,^34^ we observed that replacement of **FF** by **YF** invariantly had the same influence on biodistribution as **FY**, albeit to a slightly lower extent. In addition, **FY** showed higher affinity and selectivity for αvβ6-integrin than **YF**,^34^ which is why **FY** seemed to be generally more relevant for this type of study.

**Fig. 3:**
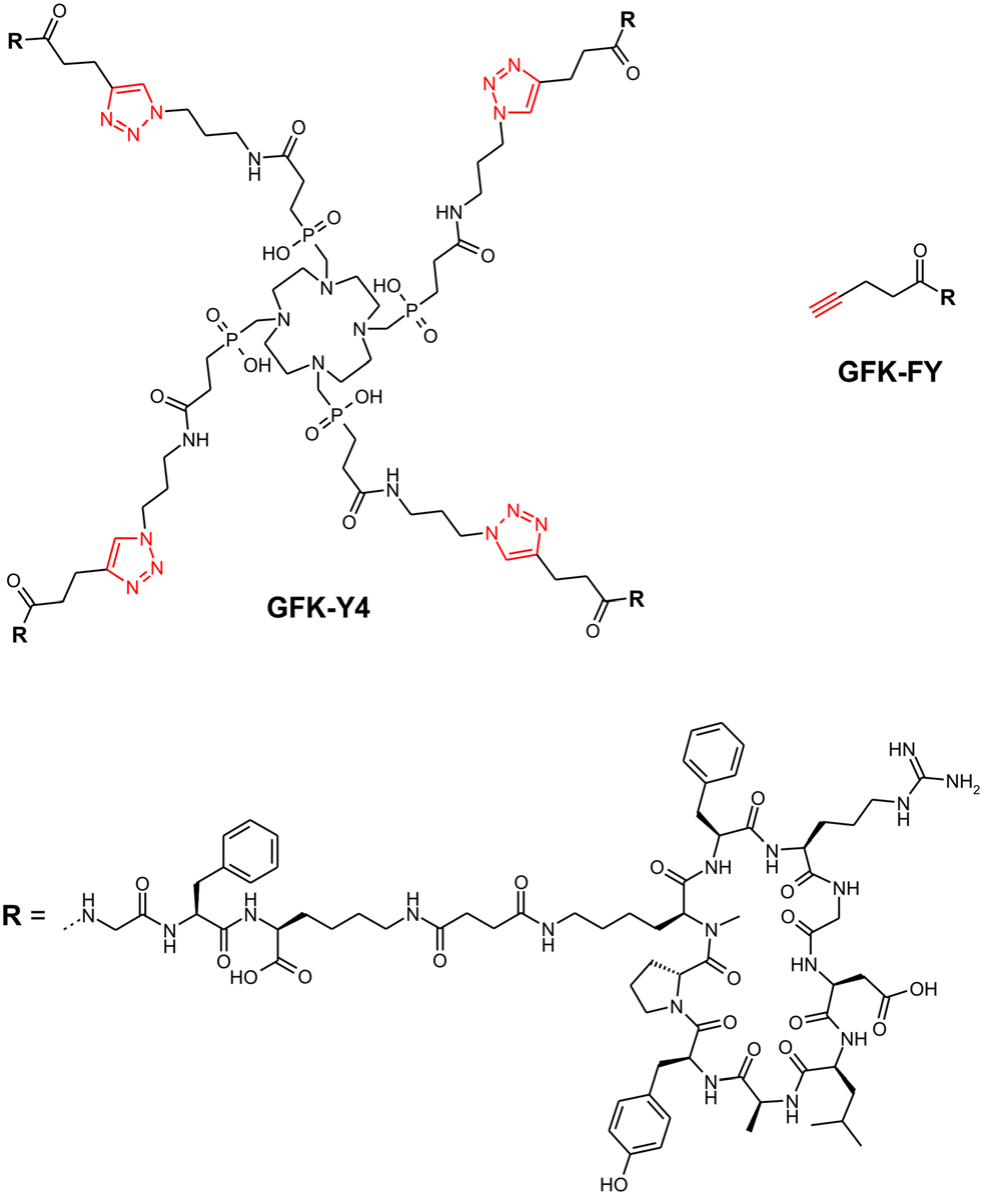
Structure of the tetramer **GFK-Y4** obtained by CuAAC of DOTPI-tetraazide with **GFK-FY**, a peptide building block comprising a mono-tyrosine cyclic peptide as featured in **FY** and the brush-border cleavable linker GFK (for a full synthesis scheme, see Experimental section). Functional groups related to the CuAAC are highlighted in red.

The synthesis of the homo-tetramers **Y0**, **Y4**, and **Y8** was done by CuAAC reactions as shown in Fig. 2, using **FF**, **FY**, and **YY**, respectively, as building blocks. **Y3** was synthesized in the same way, applying a combinatorial reaction scheme using a 1 : 3 mixture of **FF** and **FY**, after which the conjugate **Y3** was isolated by preparative HPLC. Other conjugates were not isolated. Since reaction yields were fairly low, we tested auxiliary ligands during CuAAC or alternative scavengers for demetallation for the example of the synthesis of **Y0**, and found that the yields can generally be substantially increased through optimisation if necessary. Furthermore, we were concerned about a high kidney uptake that was observed for all TRAP-trimers irrespective of the presence or absence of tyrosines.^34^ The same situation was expected for the DOTPI-tetramers, potentially leading to issues caused by a high renal radiation burden during translation. We therefore also elaborated a congener of **Y4** with tripeptide linkers between chelator and peptides, referred to as **GFK-Y4** (Fig. 3). The linker motif, Gly-Phe-Lys (GFK), has been described as stable in blood plasma, and readily cleavable by enzymes present on the brush border membrane of renal tubules.^54^ The conjugate was also assembled *via* CuAAC using a respective alkyne-functionalized building block, **GFK-FY** (Fig. 3).

### *In vivo* biodistribution and PET imaging

Radiolabeling of all conjugates was done in a straightforward manner, using n.c.a. ^177^Lu in sodium acetate buffer (pH 5.5–6), affording the radiolabeled compounds in high purities (>98% by radio-HPLC and radio-TLC; for details see Experimental section) without a separate purification step. Octanol–water distribution coefficients were determined by shake-flask and are summarized in Table 1.

**Table 1:** *N*-Octanol–water distribution coefficients at pH 7.4 (log *D*_OW_), determined by shake-flask method (*n* = 8)

Compound	Log *D*_OW_
**Y0**	−0.37 ± 0.07
**Y3**	−1.16 ± 0.23
**Y4**	−1.48 ± 0.25
**Y8**	−1.91 ± 0.20
**GFK-Y4**	−1.64 ± 0.28

**Fig. 4:**
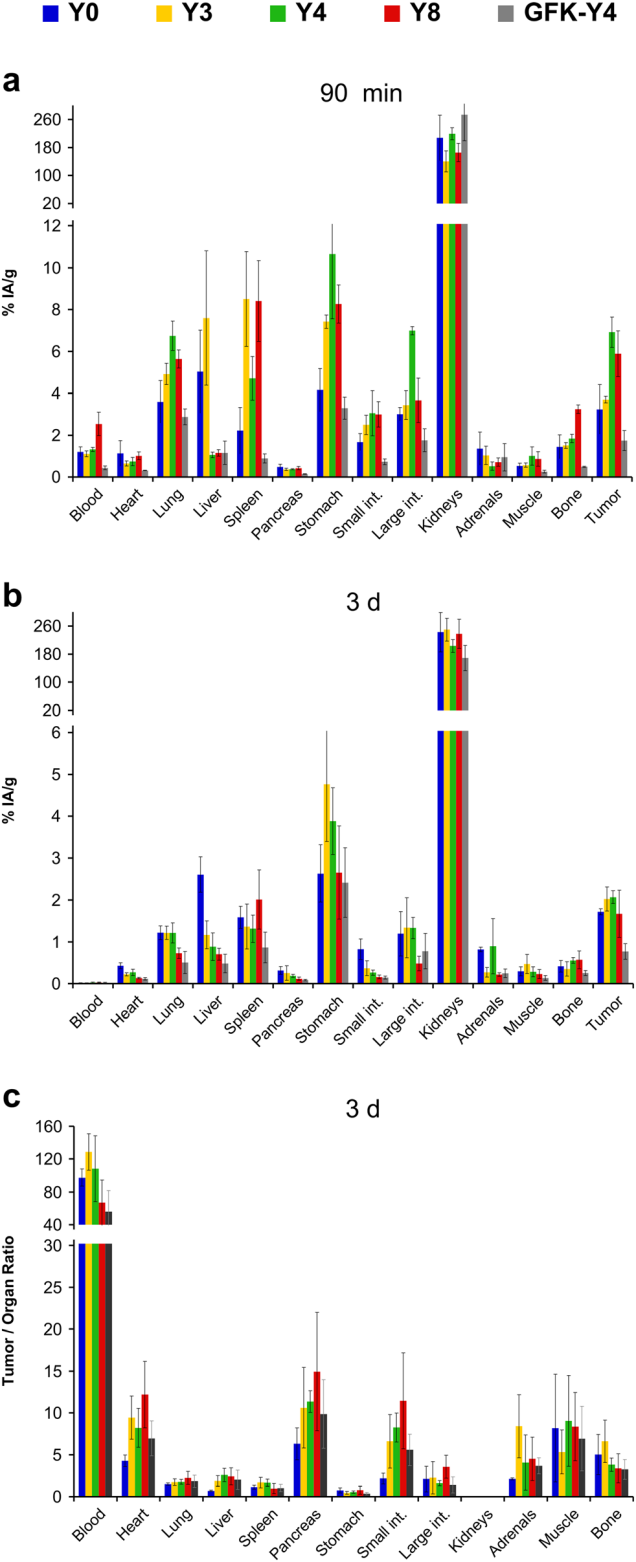
Biodistribution data for ^177^Lu-labeled peptide tetramers in H2009 (human lung adenocarcinoma) xenografted mice. For details and numerical data in tabular form, see ESI.†

The biodistribution of the ^177^Lu-labeled peptide conjugates was investigated in severe combined immunodeficiency (SCID) mice bearing subcutaneous xenografts of the αvβ6-integrin expressing human lung adenocarcinoma cell line H2009 on their right shoulders. Two representative time points after i.v. administration (p.i.) were evaluated. Uptake data were acquired 90 min p.i. to facilitate direct comparison with the respective ^68^Ga-labeled trimers,^34^ and additionally 3 d p.i. to demonstrate long-term tumor retention, which is relevant for radiotherapeutic application (Fig. 4).

For the 90 min time point, a uniform pattern for the influence of Phe → Tyr exchange on organ uptakes could not be discerned. For example, lung, stomach, intestine, and tumor uptakes were highest for ^177^Lu-**Y4**, but this compound showed the lowest uptake in the liver (Fig. 4a). Similar observations were made for the other compounds. An exception was ^177^Lu-**GFK-Y4**, whose uptakes were by far the lowest in almost all organs except the kidneys. The same situation was found 3 d p.i., with the difference that kidney activity was also lowest here (Fig. 4b). Tumor uptake of the other investigated compounds was comparable, albeit slightly in favor of ^177^Lu-**Y3** and ^177^Lu-**Y4**. The same pattern was observed for the stomach, which makes sense insofar as a physiological expression of β6-integrin in murine stomach has been confirmed earlier.^29^ The fact that tumor uptakes for ^177^Lu-**Y3** and ^177^Lu-**Y4** after 3 d were somewhat higher than those of ^177^Lu-**Y0** and ^177^Lu-**Y8** could also be caused by different administered total peptide amounts (averagely, 42 and 64 pmol for **Y3** and **Y4**, respectively, *vs.* 92 and 200 pmol for **Y0** and **Y8**, respectively; see ESI,† Tables S2, S4, S6, and S11). Although the target-specific tissue uptake of integrin ligands is usually not severely influenced by moderate changes (*i.e.*, ±50%) of injected peptide mass,^23^ a factor of approx. 4 between ^177^Lu-**Y3**/**Y4** and ^177^Lu-**Y8** might nonetheless have resulted in a relative underestimation of the tumor uptake of the latter in this study.

The tumor-to-organ ratios deserve particular attention for potential radiotherapeutics because off-target nonspecific uptakes are determining radiotoxicity and thus, clinical applicability. Again, no consistent pattern could be identified (Fig. 4c). The tumor-to-blood ratio was lowest for ^177^Lu-**Y8** among the tetramers without GFK linkers, but the same compound performed best in terms of tumor-to-pancreas and particularly tumor-to-intestines ratios. Since unwanted uptake in the intestines has proven the most critical issue hampering translation of various αvβ6-integrin targeted radiopharmaceuticals,^55^ we considered ^177^Lu-**Y8** to be the most promising compound in the series, despite the overall ambiguous data situation.

**Fig. 5:**
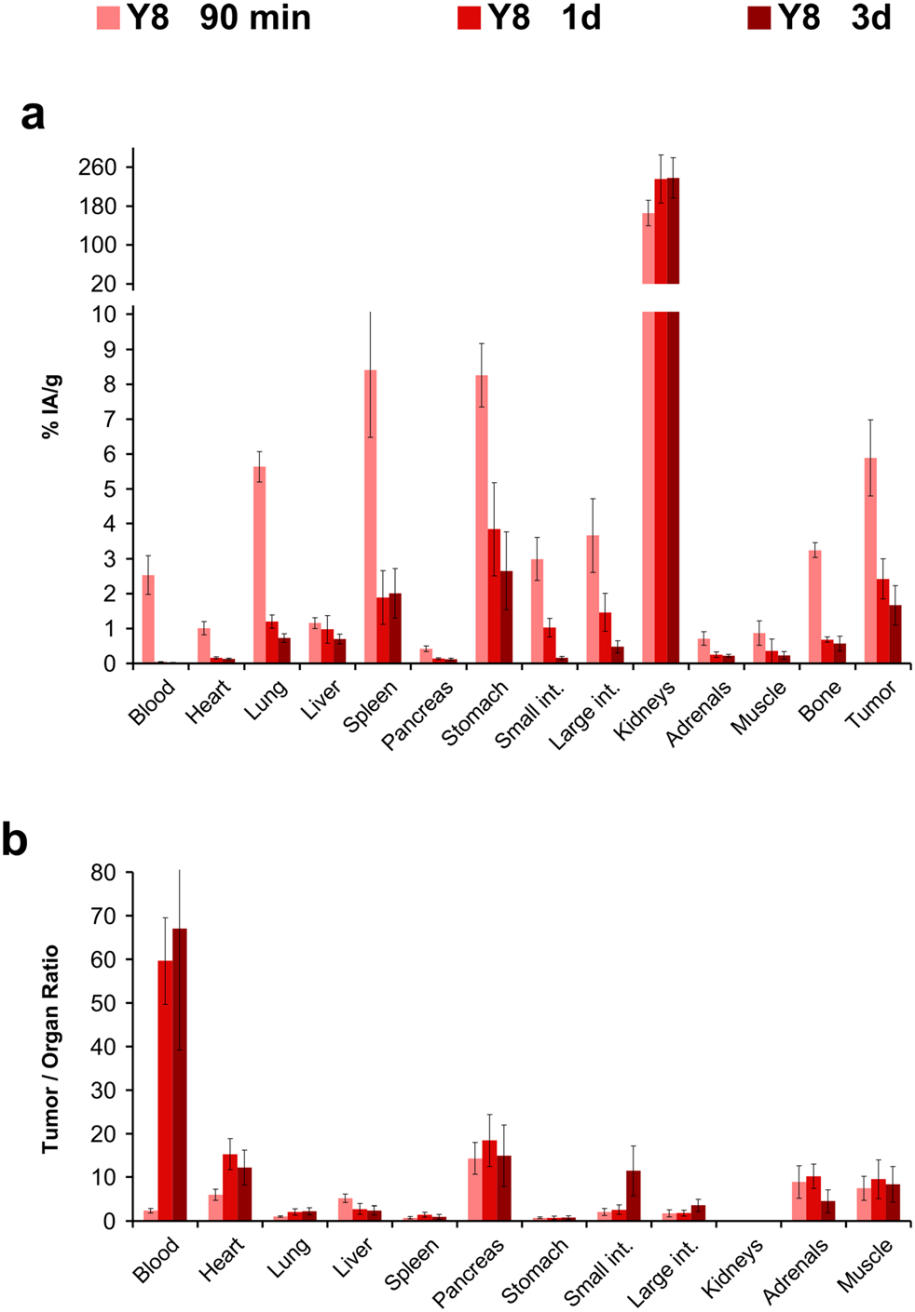
Biodistribution in H2009 tumor mice, and resulting tumor-to-organ ratios for the ^177^Lu-labeled tetramer **Y8** at different time points after injection. For details and numerical data in tabular form, see ESI.†

**Fig. 6:**
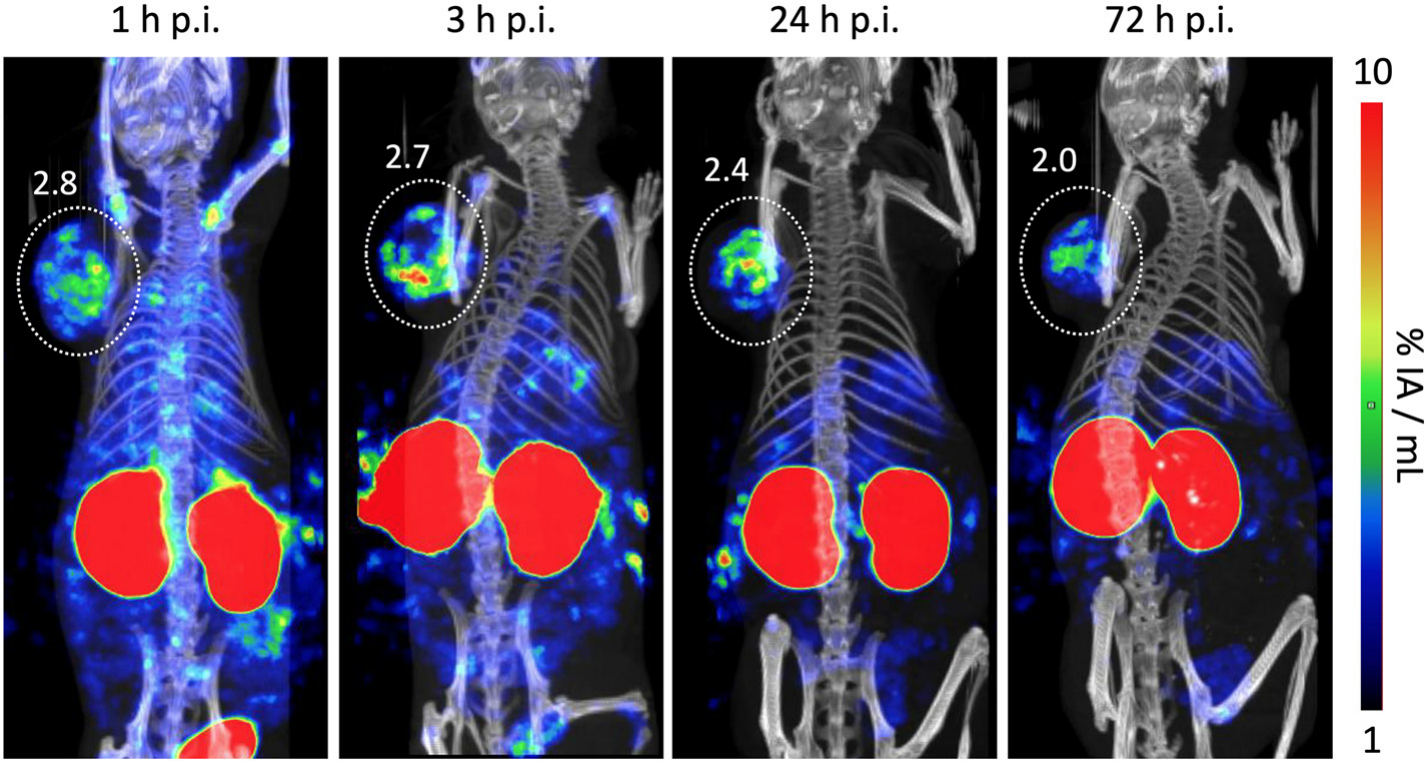
Single-photon emission computed tomography (SPECT) of a SCID mouse bearing a human H2009 tumor xenograft on the right shoulder, recorded at 4 time points (1 h, 3 h, 24 h, and 72 h) after i.v. injection of 36.5 MBq of ^177^Lu-**Y8**, scan time 1 h. Tumor position is indicated by white dotted circle. Adjacent figures denote ROI-based tumor uptakes in % IA mL^−1^.

We determined the biodistribution for ^177^Lu-**Y8** for another time point, 24 h p.i., which revealed that the compound rapidly cleared from the blood stream but was retained in many organs (Fig. 5). We noted that uptake and retention was somewhat higher for the αvβ6-integrin positive H2009 tumor and stomach and furthermore observed a similar clearance pattern, which substantiates the interpretation that the prolonged tissue accumulation was target-specific. The long-term tumor retention of ^177^Lu-**Y8** was further illustrated by single-photon emission computed tomography (SPECT) imaging (Fig. 6). The images showed an uneven tumor uptake, which corresponds well to the generally heterogeneous αvβ6-integrin expression in this model.^28,29^ Volume-of-interest (VOI) based quantification of tumor uptakes did not quite correspond to biodistribution, but nevertheless corroborated a tumor retention of ^177^Lu-**Y8** over days. However, the images also showed a high uptake in the kidneys, which did not decrease over time, consistent with the biodistribution data shown in Fig. 5a.

## Discussion

### Towards αvβ6-integrin targeted radiotherapeutics

In a series of 10 different ^68^Ga-labeled peptide trimers featuring different combinations of αvβ6-integrin targeting cyclic nonapeptides monomers of the generic formula cyclo[XRGDLAXp(*N*Me)K] (X = Phe or Tyr), the trimer comprising only tyrosines showed the lowest degree of nonspecific organ accumulation in preclinical models.^34^ This radiotracer, referred to as ^68^Ga-Trivehexin (see Fig. 1),^36^ has convincingly demonstrated its utility for PET imaging of cancers and metastases,^45^ particularly of pancreatic carcinoma.^56^ Its low intestinal uptake has proven beneficial in a clinical setting, because it resulted in a better delineation of metastases than radiotracers based on other αvβ6-integrin binding peptides.^46,55^ The next step towards improved clinical treatment of cancer patients was, of course, the development of corresponding therapeutic αvβ6-integrin radioligands to enable a theranostic approach.^46,57^

We previously evaluated the potential of various ^177^Lu labeled 1 : 1 chelator–peptide conjugates of cyclo[FRGDLAFp(*N*Me)K] but found that such monomers are generally not suitable for radioligand therapy due to low uptake and retention in tumors (<0.8% IA g^−1^, 90 min p.i.).^58^ The novel peptide tetramers ^177^Lu-**Y0**, ^177^Lu-**Y3**, ^177^Lu-**Y4**, and ^177^Lu-**Y8** showed a markedly improved tumor uptake (range 3.2 to 6.9% IA g^−1^, 90 min p.i.) and a retention over days (range 1.7 to 2.1% IA g^−1^, 3 d p.i.; see Fig. 4, Fig. 6, and ESI† Tables S1–S11). However, one cannot fail to notice a high and persistent kidney uptake of these agents, which is expected to limit their clinical applicability because of a high risk of radiation-induced renal damage.^59^ This problem has been observed for many radiolabeled peptides and proteins, and is most likely caused by a long residence time of the radiometabolites generated by lysosomal proteolysis of the radiopeptides in renal tubular cells.^60^

Different approaches have been pursued to solve this issue. One strategy aims at selective breakdown of radiopharmaceuticals that have accumulated in the kidneys. This can be achieved by introducing peptidic linkers that are stable in blood plasma but are cleaved by enzymes present on the brush border membrane of renal tubules, to selectively generate small radiometabolites in the kidneys that are rapidly excreted *via* the urine.^61^ One such linker is the amino acid sequence GFK that is recognized by neutral endopeptidase (NEP) located in the renal brush border.^54^ We consequently equipped one of the tetramers, **Y4**, with this sequence in all peptide–chelator linkages, resulting in the conjugate **GFK-Y4** (Fig. 3). Unfortunately, the approach did not result in the expected substantial improvement of the tumor-to-kidney ratio. The renal retention of ^177^Lu-**GFK-Y4** was still high after 3 d, and the compound furthermore showed lower tumor uptake and faster elimination from the blood stream than the other tetramers (Fig. 4). Such an *in vivo* behavior suggests that the GFK sequence is actually cleaved before reaching the brush border, leading to premature decomposition and excretion of a large fraction of the radiopharmaceutical before it can bind to its target on the tumor cells. The desired reduction in renal uptake while maintaining tumor uptake could therefore not be achieved in this explorative experiment. Further linker variations or other established methods to reduce the radiation dose of the kidneys, such as the simultaneous infusion of renal protection agents,^62^ shall therefore be evaluated in the future.

### Structure, polarity, and biodistribution revisited

**Fig. 7:**
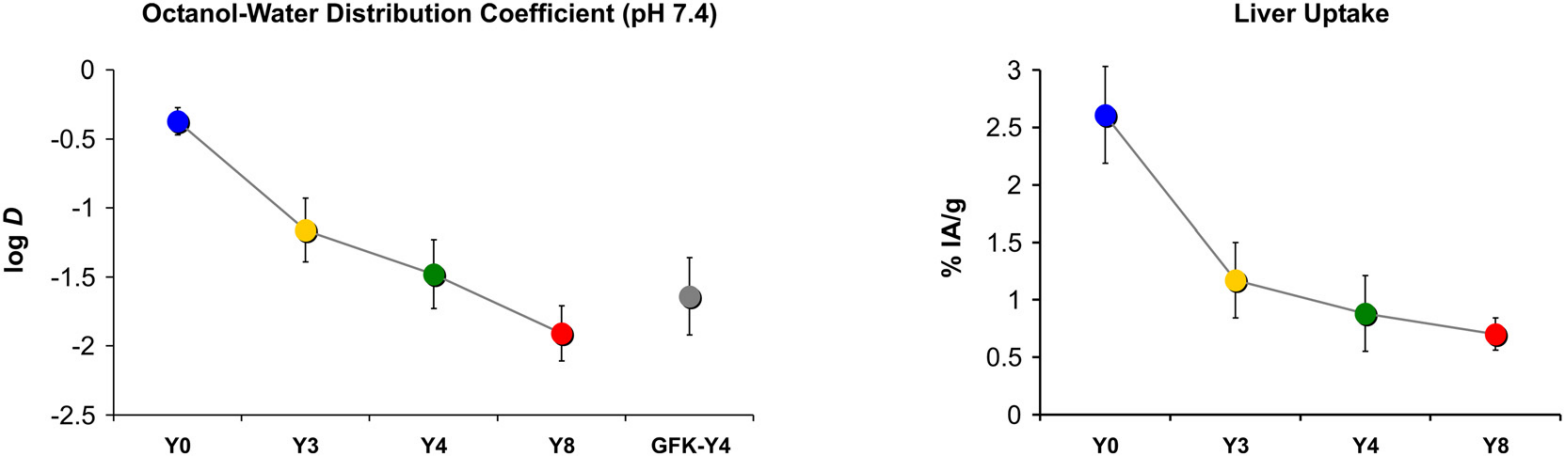
Comparison of trends in polarity proxies and liver uptakes, 3 d p.i. Connecting lines between data points are intended only to visualize trends and do not indicate a functional correlation.

The other aim of this study was to figure out whether multimers of cyclo[XRGDLAXp(*N*Me)K] (X = F or Y) that are based on another scaffold than TRAP show the same surprising trends of polarity measures, especially log *D*, and non-specific organ uptakes, especially in the liver. We recall that for the trimer series, replacement of less than 50% of phenylalanines by tyrosines did not change the log *D* but reduced liver uptake by up to 72%.^34^ Such an observation was not made for the tetramers of this work (Fig. 4). We found a consistent decrease of both parameters with gradual Phe → Tyr exchange. The first 3 Phe → Tyr substitutions (**Y0** → **Y3**) still had a larger influence on both parameters than the last 4 exchanges (**Y4** → **Y8**), but the overall picture is well in line with the textbook scheme, *i.e.*, a higher degree of polarity reduces liver absorption (Fig. 7).

**Fig. 8:**
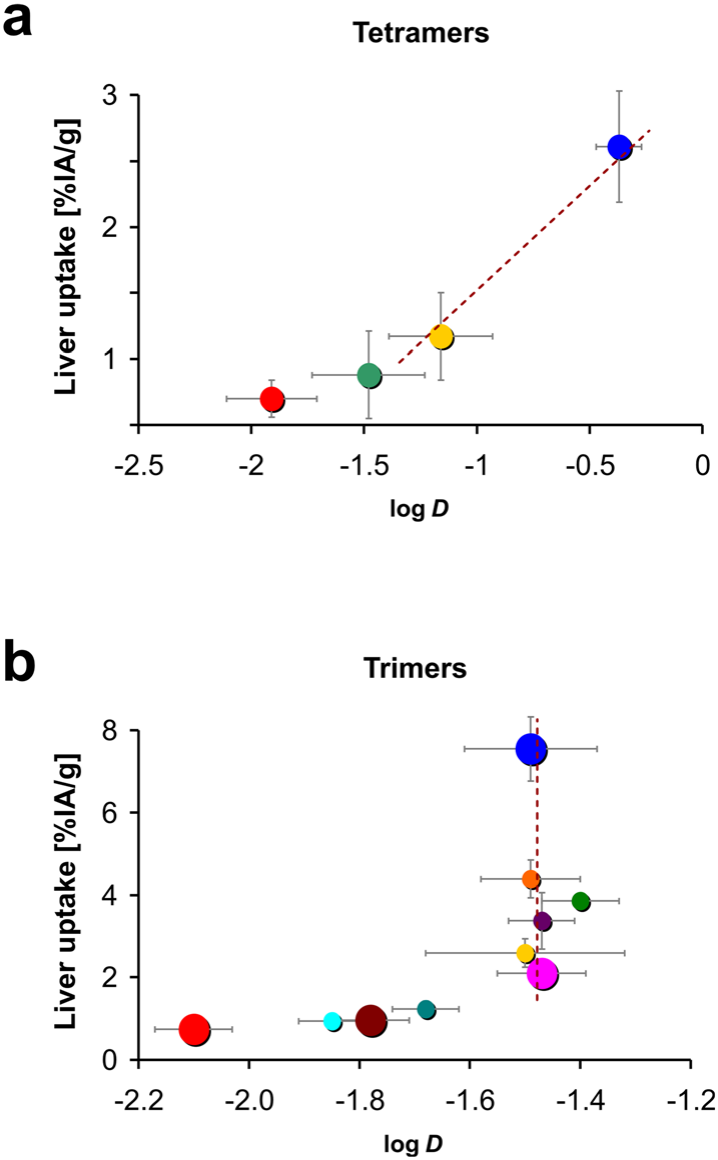
Plots of liver uptakes against octanol–water distribution coefficients (log *D*) for a) peptide tetramers (this work) and b) Trivehexin-related trimers reported earlier (*RSC Med. Chem.* 2023, **14**, 2429).^34^ Large-size circles in b highlight the conjugates of the trimer series (**Y0**, **Y2F**, **Y3F**, and **Y6**, see ref. 34, Fig. 1) which, in terms of percentage and distribution of Phe/Tyr units, are most equivalent to the four tetramers in this work. Color codes of circles correspond to the ones used for the respective compound series; for trimers see ref. ^34^, Fig. 2a; for the tetramers, see this work, Fig. 4. Note that similar colors in a and b do not necessarily indicate a structural match.

Fig. 8 further illustrates that the tetramer series did not show the anomaly that was previously observed for the corresponding library of TRAP-based trimers, namely, that polarity proxies such as log *D* had no predictive value for certain *in vivo* properties, such as liver uptake.^34^ For the tetramers, an increase of the log *D* always corresponded to an increase of liver uptake (Fig. 8a). It thus appears possible to derive a bijective correlation function, which could be used to approximate the liver uptake of a novel compound in that series based on its log *D* (exemplified by the dotted line in Fig. 8a). In lack of a suitable theory, we are however not able to define which type of regression could be appropriate to obtain a fit function for a correlation between the logarithm of the octanol–PBS distribution coefficient and the liver uptake expressed as % IA g^−1^. In contrast, a fit was not meaningful at all for the trimers,^34^ which is particularly evident from the fact that in the corresponding plot (see Fig. 8b), five out of ten data points for the trimer series are more or less vertically aligned. A hypothetical correlation function would therefore have to comprise non-bijective sections (indicated by a vertical dotted line in Fig. 8b), which means it does not exist.

Taken together, we found that in contrast to αvβ6-integrin binding cyclic RGD peptide trimers,^34^ the tetramers behaved “normally”, *i.e.*, the relationship between polarity and liver absorption adhered to the textbook pattern. Their conformational structure apparently does not promote a synergistic interaction between certain structural elements or the peptide monomers that would lead to non-linear effects in this regard. Therefore, it seems plausible to assume that for the DOTPI conjugates, *in vitro* polarity proxies such as log *D* may be more useful to guide early-stage drug development than in case of TRAP trimers.

### The importance of tyrosines in peptide multimers

Regardless of whether or not a synergistic interaction of phenylalanines caused a disproportionately high non-specific organ uptake, one fundamental observation made for both groups of cyclopeptide multimers studied (^68^Ga-TRAP trimers or ^177^Lu-DOTPI tetramers) was the same: more tyrosines improved the *in vivo* performance. In particular, undesired non-target uptake was reduced, while target-specific binding *in vivo* (*i.e.* tumor uptake) of the conjugates remained more or less unchanged or was even improved. Similar findings have already been forged into a golden rule for the development of synthetic binding proteins. Koide and Sidhu pointed out in 2009 that non-natural engineered proteins showed better binding properties if they consisted predominantly of Tyr, Ser, and Gly.^63^ These authors described the unique role of Tyr as follows: “The physicochemical properties of tyrosine make it the amino acid that is most effective for mediating molecular recognition, and protein engineers have taken advantage of these characteristics to build tyrosine-rich protein binding sites that outperform natural proteins in terms of affinity and specificity.” However, the same concept does not seem to play a major role for peptides, as there is apparently no systematic tendency to favor tyrosine-containing sequences over comparable peptides without Tyr. To stay with integrin ligands, even a glance into the wealth of RGD peptide literature^8,57^ quickly reverals that there is apparently no clear preference for either of the two most popular αvβ3-integrin binding motifs, c(RGDyK) and c(RGDfK), although the former contains a d-Tyr and the latter does not.

With regard to the present work, it should be mentioned that during the development of the nonapeptides used herein, the sequence cyclo[FRGDLAFp(NMe)K] showed a slightly better αvβ6-integrin affinity and subtype selectivity than its two siblings with one Phe replaced by a Tyr.^64^ Therefore, it was initially chosen as a lead structure for radiopharmaceutical development.^29,58^ However, as long as such peptide monomers are not directly used as drugs (either non-functionalized or equipped with simple tags) but as components of larger constructs, it seems reasonable not to rely exclusively on the “best” peptide, but also to consider their “second best” congeners as long as they contain more tyrosines. Our data convincingly demonstrated that it can be advantageous to follow this rationale in the development of radiopharmaceuticals, as our multimers comprising only cyclo[FRGDLAFp(*N*Me)K] were outperformed by those with tyrosines, although all corresponding Tyr-containing monomers exhibited less favorable *in vitro* affinities and selectivities.^34^

## Conclusion

The overarching message of our two multimerization studies (*RSC Med. Chem.,* 2023, **14**, 2429,^34^ and this work) is that the development of peptide multimers should preferably be guided by the concepts and rules known from protein engineering. This is because peptide multimers are not simply somewhat larger molecules whose properties are the sum of the characteristics of the monomeric peptide components of which they are composed. Rather, they should be perceived as a special class of small or medium-sized proteins, which have their own special design rules that need to be systematically explored. We conclude that the performance of multimers is not primarily determined by the *in vitro* properties (especially affinity and selectivity) of the peptide monomers, but rather on the peptides' suitability for the overall macromolecular design concept. One lesson we have learned is that tyrosines in the peptide sequence can contribute a great deal to this suitability. This particular aspect has proven to be crucial for the development of αvβ6 integrin-targeted radiopharmaceuticals.

## Experimental Section

### Materials & methods

Analytical HPLC was performed on a Shimadzu (Kyoto, Japan) Prominence HPLC system equipped with either a Dr. Maisch ReproSil Pur C18 AQ 150 × 4.6 mm, 5 μm, 120 Å (AC1) or a Phenomenex Kinetex C18 150 × 4.6 mm 5 μm column (AC2). Semipreparative HPLC was performed on a Shimadzu HPLC system equipped with either a Dr. Maisch ReproSil Pur C18 AQ 250 × 10 mm, 5 μm, 120 Å column with 5 mL min^−1^ flow (PC1) or a Dr. Maisch ReproSil Pur C18 150 × 30 mm, 5 μm, 120 Å column with 20 mL min^−1^ flow (PC2). Solvents for both systems were water (A) and acetonitrile (B) each supplemented with 0.1% (vol) trifluoroacetic acid (TFA). ESI-MS spectra (positive mode) were recorded on a Shimadzu LC-MS 2020 single quadrupol mass spectrometer. Solvents for MS applications were water (A) and acetonitrile (B) each supplemented with 0.1% (vol) formic acid. The other experimental procedures (determination of log *D*_7.4_, cultivation of H2009 human lung adenocarcinoma cells, generation of tumor xenografts in CB17 SCID mice, and biodistribution) were performed as described before.^28^ All animal experiments have been carried out according to applicable law and institutional guidelines of Technical University of Munich, and were approved by the responsible local authority (Regierung von Oberbayern). The H2009 cells were regularly authenticated and tested for mycoplasma contamination. DOTPI-tetraazide,^43^**FF** (previously termed AvB6),^29^**FY**,^34^ and **YY** (also referred to as Tyr2-alkyne)^36^ were synthesized as described previously.

### Syntheses

**Fig. 9:**
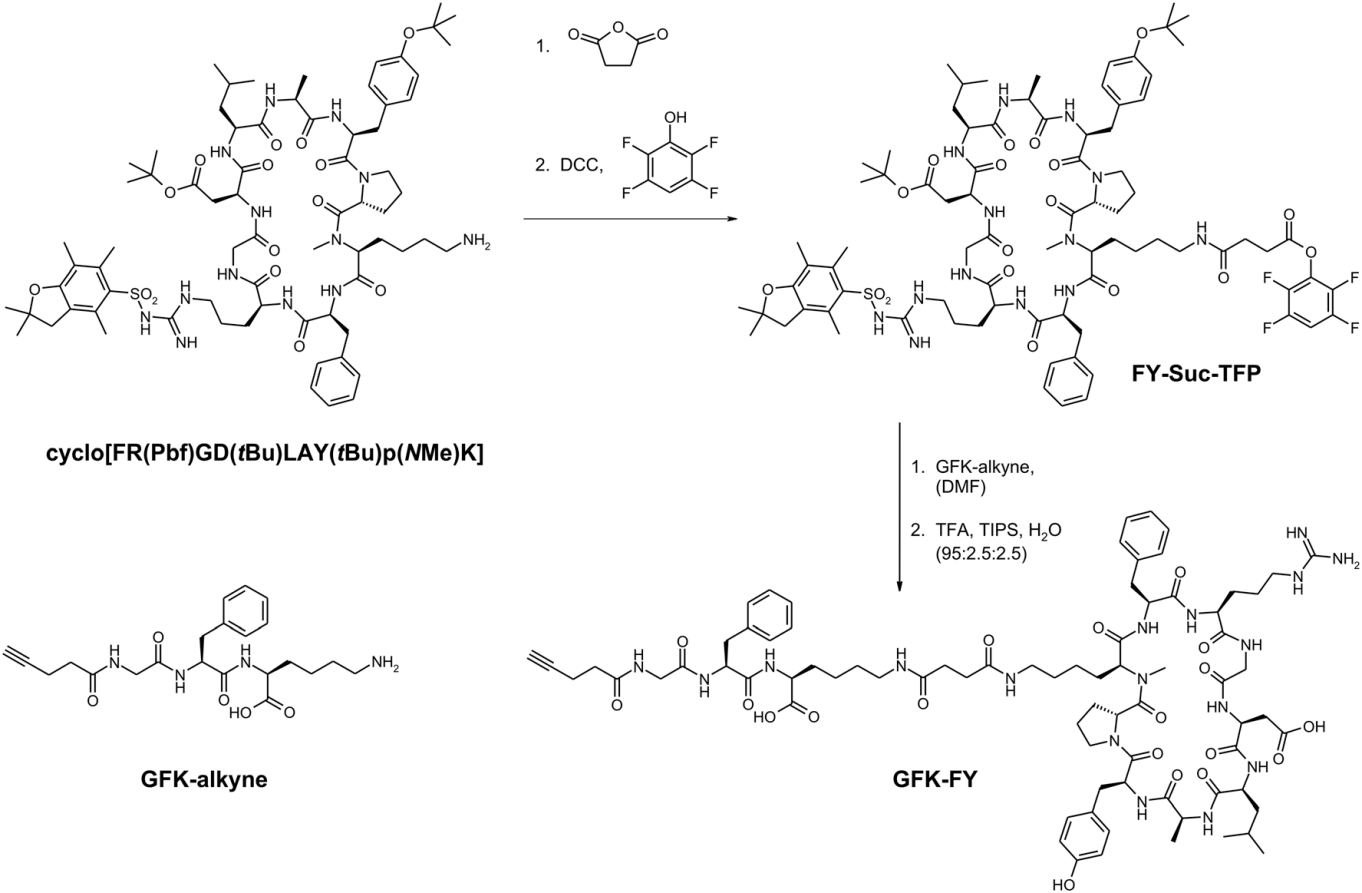
Synthesis of the building block **GFK-FY**, a derivative of **FY** featuring the brush border cleavable linker GFK.

The synthesis of the **Y4** analog GFK-linker functionalized peptide building block **GFK-Y4** is outlined in Fig. 9. Corresponding chromatograms and spectra are given in the ESI.†

#### FY-Suc-TFP

The protected cyclic peptide cyclo[FR(Pbf)GD(*t*Bu)LAY(*t*Bu)p(*N*Me)K]^34^ was reacted with succinic acid anhydride according to a published procedure.^65^ 143 mg (111 μmol, 1.0 eq.) of peptide were dissolved in a small amount of dry dichloromethane (DCM) and 12.2 mg (122 μmol, 1.1 eq.) of succinic anhydride were added. The mixture was cooled to 0 °C and triethylamine (16.9 μL, 122 μmol, 1.1 eq.) was added slowly. The ice bath was removed and the reaction was allowed to run over night with stirring. For workup DCM was removed *in vacuo* and the crude residue was dissolved in H_2_O/MeCN, followed by purification *via* semipreparative HPLC (20–60% B in 15 min, PC2). After lyophilization, **FY-Suc** was obtained as a colorless solid (89%, 150 mg, 100 μmol). This intermediate was dissolved in dimethyl formamide (DMF) and 33 mg (33 μmol, 2.0 eq.) of 2,3,5,6-tetrafluorophenol and 21 mg (100 μmol, 2.0 eq.) of dicyclohexyl carbodiimide (DCC) were added to the solution. The product was purified *via* semi-preparative HPLC (20–60% B in 15 min, PC2) and lyophilized, affording a colorless solid (90%, 150 mg, 90 μmol). *m*/*z*: 1675.7 [M + H^+^]^+^, 838.4 [M + 2H^+^]^2+^**FY-Suc-TFP** RP-HPLC (10–90% B in 15 min, AC2): 11.5 min.

#### GFK-alkyne (brush-border cleavable linker)

The GFK-motif was assembled on a CTC-resin. First, Fmoc-l-Lys(Dde)-OH (1.3 g, 2.1 mmol, 1.0 eq.) was loaded on to the resin using 1.1 mL (3.0 eq.) of DIPEA in 20 mL of DMF. After subsequent Fmoc-deprotection, Fmoc-l-Phe-OH (1.6 g, 4.1 mmol, 2.0 eq.) was coupled to the free amine using 1.6 g (4.1 mmol, 2.0 eq.) of HATU, 0.6 g (4.1 mmol, 2.0 eq.) of 1-hydroxybenzotriazol (HOBt) and 1.9 mL (10.3 mmol, 5.0 eq.) of diisopropyl ethylamine (DIPEA). Next, Fmoc-Gly-OH (1.2 mg, 4.1 mmol, 2.0 eq.) was reacted with the unprotected *N*-terminus using the same conditions as mentioned above. Lastly, the Fmoc-deprotected tripeptide was functionalized with 4-pentynoic acid (0.4 g, 4.1 mmol, 2.0 eq.), followed by Dde-deprotection with 5% hydrazine in DMF. GFK-alkyne was cleaved from the resin using a cocktail of trifluoroacetic acid (TFA)/triisopropyl silane (TIPS)/H_2_O (95 : 2.5 : 2.5) (3 × 4 mL). After removal of all volatiles *in vacuo*, the residue was purified *via* semipreparative HPLC (10–40% B in 15 min, PC2) and subsequently lyophylized (50%, 430 mg, 1 mmol). *m*/*z*: 431.3 [M + H^+^]^+^, 453.3 [M + Na^+^]^+^. **GFK-alkyne** RP-HPLC (10–90% B in 15 min, AC2): 6.0 min.

#### GFK-FY (Fig. 9)

**GFK-alkyne** (15.0 mg, 33 μmol, 1.0 eq.) was reacted with **FY-Suc-TFP** (84 mg, 50.2 μmol, 1.5 eq.) in DMF (30 mL). The pH of the solution was adjusted to 8–9 with DIPEA (70 μL, 8.0 eq.) and the mixture was stirred at room temperature for 16 h. All volatiles were removed *in vacuo* and the crude residue was treated with a cocktail of TFA/TIPS/H_2_O (95 : 2.5 : 2.5) (3 mL) for final deprotection. TFA was removed under a stream of air and the peptide was purified *via* semipreparative HPLC (20–60% B in 15–min, PC2). After lyophylization GFK-FY was obtained as a colorless solid (85%, 44.1 mg, 28 μmol). *m*/*z*: 1576.1 [M + H^+^]^+^, 788.5 [M + 2H^+^]^2+^. **GFK-FY** RP-HPLC (10–90% B in 15 min, AC2): 6.2 min.

#### General procedure for synthesis of DOTPI-tetramers

The synthesis was modified from the literature.^43^ 4.4 eq. of the respective peptide building block (a mixture was used for synthesis of **Y3**) was added to a solution of DOTPI-tetraazide (1.0 eq.) and copper(ii) acetate hydrate (2.0 eq.) in a minimum amount of H_2_O or a mixture of H_2_O and *t*BuOH to aid dissolution of reagents. Sodium ascorbate (50.0 eq.) dissolved in a minimum volume of H_2_O was quickly added to the reaction, the mixture vortexed for 1 min, and placed in a water bath at 60 °C. Upon addition of sodium ascorbate to the reaction mixture, a blue precipitate formed slowly which dissolved after 2–5 min, resulting in a transparent dark blue solution. The reaction was allowed to progress for 1 h, followed by monitoring reaction progress by HPLC-MS. Cu species were sequestered from the tetramer and the reaction solution by addition of NOTA (30.0 eq.) dissolved in H_2_O with adjustment of the pH to 2.2 using concentrated 12 M HCl (typically 5–10 μL). The Cu transchelation reaction was allowed to progress for 1 h at 60 °C. The metal-free tetramers were then directly purified by RP-HPLC, followed by lyophilization. Corresponding chromatograms and MS spectra are given in the ESI.†

#### Y0

This compound was first synthesized according to the general procedure with 15.1% overall yield. We later tested alternative reaction conditions and found that an optimization of the yield is feasible: FF*TFA (8.18 mg, 6.6 μmol) and DOTPI-tetraazide*2TFA (2.0 mg, 1.5 μmol) in 500 μL H_2_O/*t*BuOH (1 : 1). Copper(ii) acetate hydrate (1.49 mg, 7.5 μmol), sodium ascorbate (17.8 mg, 90 μmol) and tris(3-hydroxypropyltriazolylmethyl)amine (THPTA, 0.33 mg, 0.75 μmol) in 500 μL H_2_O/*t*BuOH (1 : 1). Catalyst solution was added to azide/alkyne solution and the mixture was stirred for 10 min at room temperature. Cu species were demetallated as described above with NOTA (6.82 mg, 22.5 μmol) and bispidol (2.68 mg, 4.5 μmol) in 1 mL H_2_O/ACN pH 2.4 for 1 h at 60 °C. **Y0** was obtained as the TFA salt in form of a colorless solid (46.7%, 4.0 mg, 0.70 μmol). *m*/*z*: 1403.1 [M + 4H^+^]^4+^, 1122.5 [M + 5H^+^]^5+^, 935.3 [M + 6H^+^]^6+^. ^177^Lu-**Y0** RP-HPLC (0–99% B in 15 min, AC1): *t*_R_ = 9.4 min.

#### Y3

Reagents: **FF** (1.2 eq., 5.4 μmol) **FY** (3.6 eq., 16.2 μmol) DOTPI tetraazide (5.0 mg, 4.5 μmol, 1.0 eq.) in 350 μL of H_2_O/*tBu*OH (1 : 2), Cu^II^ acetate hydrate (2.0 mg, 10 μmol, 2.2 eq.), Na ascorbate (36.1 mg, 182 μmol, 40.0 eq.). A Cu^II^ complex of **Y3** was isolated from the CuAAC reaction mixture by preparative HPLC (32–48% B in 20 min, PC1). Afterwards, Cu species were demetallated as described above, followed by final purification by HPLC. (35% B isocratic, PC1). After lyophilization **Y3** was obtained as the TFA salt in form of a colorless solid (1.7%, 450 μg, 78 nmol). *m*/*z*: 1886.0 [M + 3H^+^]^3+^, 1414.8 [M + 4H^+^]^4+^, 1132.1 [M + 5H^+^]^5+^, 943.6 [M + 6H^+^]^6+^. ^177^Lu-**Y3** RP-HPLC (0–99% B in 15 min, AC1): *t*_R_ = 8.6 min.

#### Y4

Reagents: **FY** (10.49 mg, 9.18 μmol), DOTPI-tetraazide (2.3 mg, 2.09 μmol), copper(ii) acetate hydrate (833 μg, 4.18 μmol) and sodium ascorbate (20.67 mg, 103.32 μmol) in 410 μL H_2_O. Cu species were demetallated as described above with NOTA (16.71 mg 55.09 μmol) in 1 mL H_2_O. **Y4** was obtained as the TFA salt in form of a colorless solid (13%, 1.35 mg, 0.23 μmol). *m*/*z*: 1891.4 [M + 3H^+^]^3+^, 1418.8 [M + 4H^+^]^4+^, 1135.3 [M + 5H^+^]^5+^, 946.3 [M + 6H^+^]^6+^. ^177^Lu-**Y4** RP-HPLC (0–99% B in 15 min, AC1): *t*_R_ = 8 min.

#### Y8

Reagents: (9.43 mg, 8.29 μmol), DOTPI-tetraazide (2.07 mg, 1.88 μmol), copper(ii) acetate hydrate (752 μg, 3.77 μmol) and sodium ascorbate (18.66 mg, 94.18 μmol) in 345 μL H_2_O. Cu species were demetallated as described above with NOTA (16.71 mg, 55.09 μmol) in 1 mL H_2_O. **Y8** was obtained as the TFA salt in form of a colorless solid (34.5%, 3.71 mg, 0.6 μmol). *m*/*z*: 1434.56 [M + 4H^+^]^4+^, 1147.75 [M + 5H^+^]^5+^, 956.77 [M + 6H^+^]^6+^. ^177^Lu**-Y8** RP-HPLC (0–99% B in 15 min), AC1): *t*_R_ = 8.4 min.

#### GFK-Y4

Reagents: **GFK-FY** (6.26 mg, 3.98 μmol), DOTPI-tetraazide (972 μg, 0.88 μmol), copper(ii) acetate hydrate (752 μg, 3.77 μmol) and sodium ascorbate (18.66 mg, 94.18 μmol) in 540 μL H_2_O/*tBu*OH (1 : 1.7). Cu species were demetallated as described above with NOTA (7.02 mg, 23.15 μmol) in 0.5 mL H_2_O. **GFK-Y4** was obtained as the TFA salt in form of a colorless solid (22.4%, 1.28 mg, 0.17 μmol). *m*/*z*: 1480.52 [M + 5H^+^]^5+^, 1234.00 [M + 6H^+^]^6+^, 1057.71 [M + 7H^+^]^7+^. ^177^Lu-**GFK-Y4** RP-HPLC (0–99% B in 15 min, AC1): *t*_R_ = 8.1 min.

### Radiolabeling

Labeling of the tetramers with ^177^Lu was performed manually in 300 μL of a 1 M NaOAc/HOAc-buffer (pH 5.9). 1–7 nmol (1–7 μL of an 1 mM stock solution) of labeling precursor were incubated with 5–106 μL (32–106 MBq) of ^177^LuCl_3_ in 0.04 M HCl (Endolucin Beta, ITM, Germany) for 15–60 min at 95 °C. Completion of the reaction was monitored with radio-TLC or radio-HPLC (for chromatograms see ESI†).

### SPECT imaging

SPECT imaging was performed for 60 min per animal on a nanoScan SPECT/CT (Mediso, Budapest, Hungary) at different timepoints (1 h, 3 h, 24 h, 72 h) after intravenous injection of approx. 40 MBq [^177^Lu]Lu-**Y8** per animal (*n* = 2). Reconstruction, image analysis and quantification of SPECT data were performed using Nucline and Interview fusion software (both Mediso).

## Conflicts of interest

N. G. Q. and J. N. are inventors on patent applications related to αvβ6-integrin binding peptide conjugates and ^68^Ga-Trivehexin. J. N. and J. Š. are CSO and CEO, respectively, and co-founders of TRIMT GmbH (Radeberg, Germany) who has licensed IP from TU Munich. J. N. is furthermore a member of the Scientific Advisory Board of Radiopharm Theranostics LLC (Carlton, Australia) who has licensed IP from TRIMT GmbH. S. K. receives research support from TRIMT GmbH.

## Supplementary Material

MD-015-D4MD00073K-s001
