# Supplementary material for: The importance of tyrosines in multimers of cyclic RGD nonapeptides: towards αvβ6-integrin targeted radiotherapeutics
Source: RSC Med Chem. 2024 Apr 19;15(6):2018–29. doi: 10.1039/d4md00073k (PMC11187563; doi:10.1039/d4md00073k)
Supplement: MD-015-D4MD00073K-s001 [file MD-015-D4MD00073K-s001.pdf]

Electronic Supplementary Material (ESI) for RSC Medicinal Chemistry.
This journal is © The Royal Society of Chemistry 2024

Supplemental Information

The importance of tyrosines in multimers of cyclic RGD nonapeptides: towards αvβ6-integrin targeted radiotherapeutics

Neil Gerard Quigley, Maximilian Alexander Zierke, Beatrice Stefanie Ludwig, Frauke Richter, Nghia Nguyen, Falco Reissig, Jakub Šimeček, Susanne Kossatz*, Johannes Notni*

1. HPLC and ESI-MS data

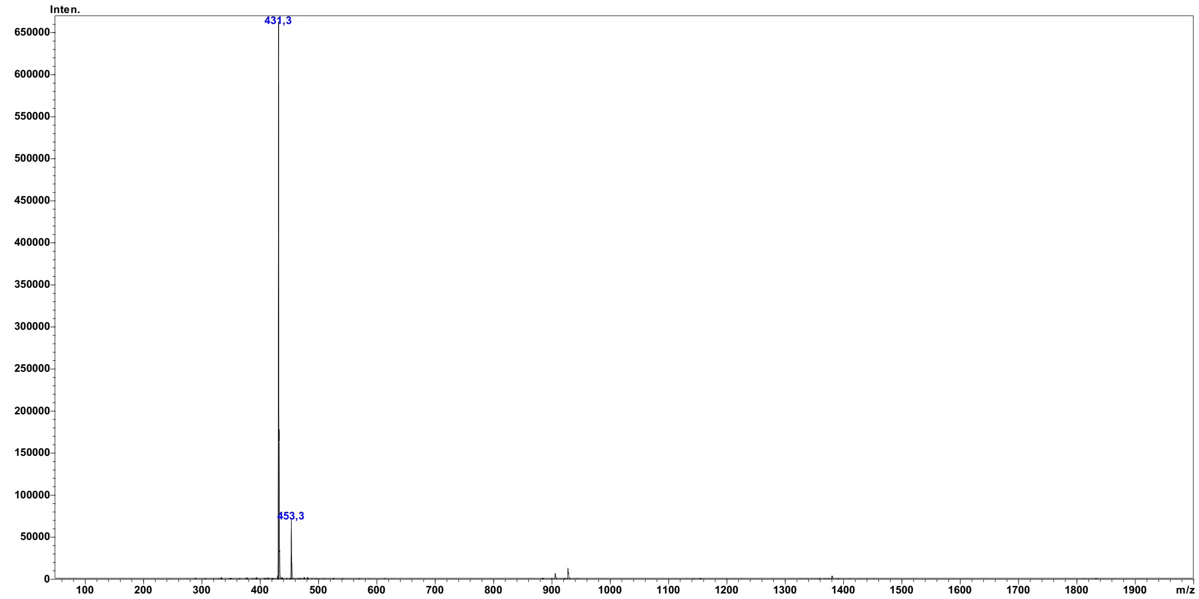

Figure S1. ESI-MS of **GFK-alkyne**; m/z corresponds to molecular composition as follows: 431.3 $[M+H^+]^+$, 453.3 $[M+Na^+]^+$.

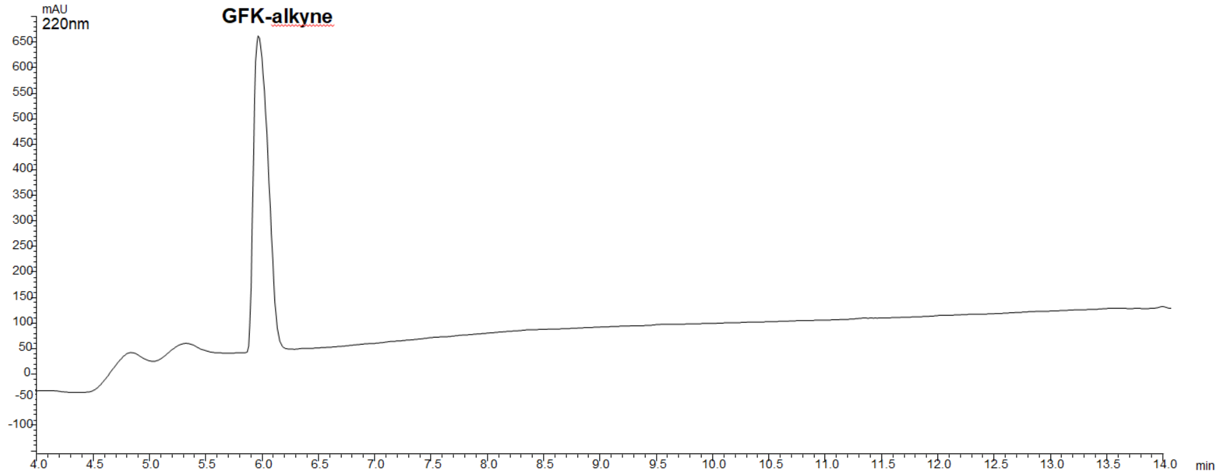

Figure S2. RP-HPLC of **GFK-alkyne** using analytical column AC2; 0.5 mL/min; gradient: 10–90% B in 15 min; t_R = 6.0 min.

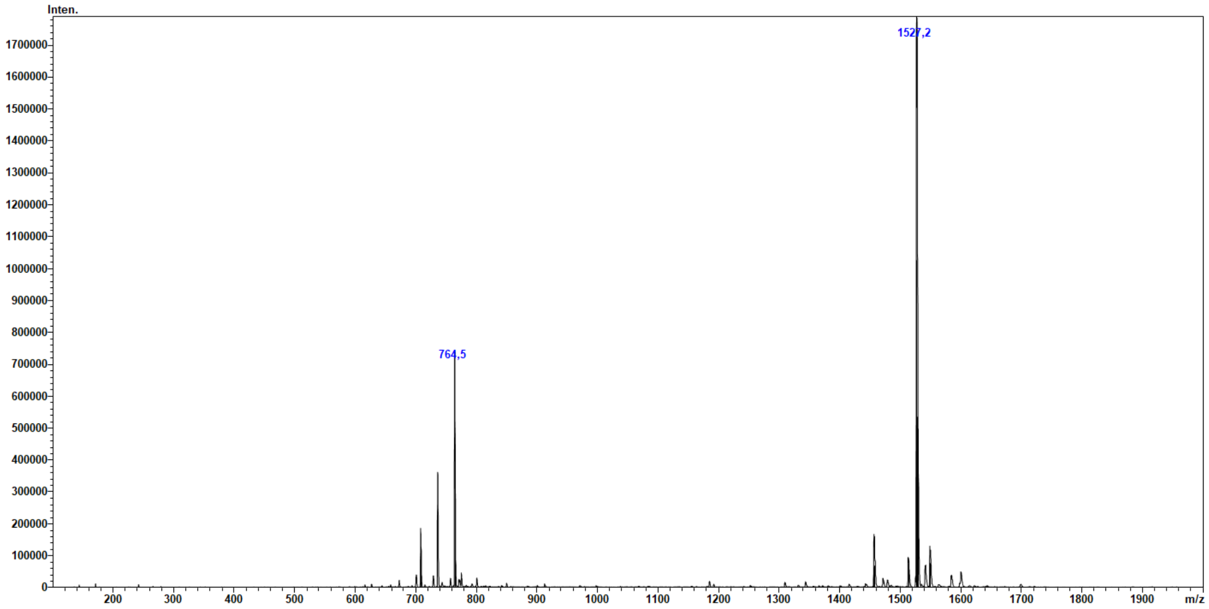

Figure S3. ESI-MS of **FY-Suc**; m/z corresponds to molecular composition as follows: 1527.2 $[M+H^+]^+$, 764.5 $[M+2H^+]^{2+}$.

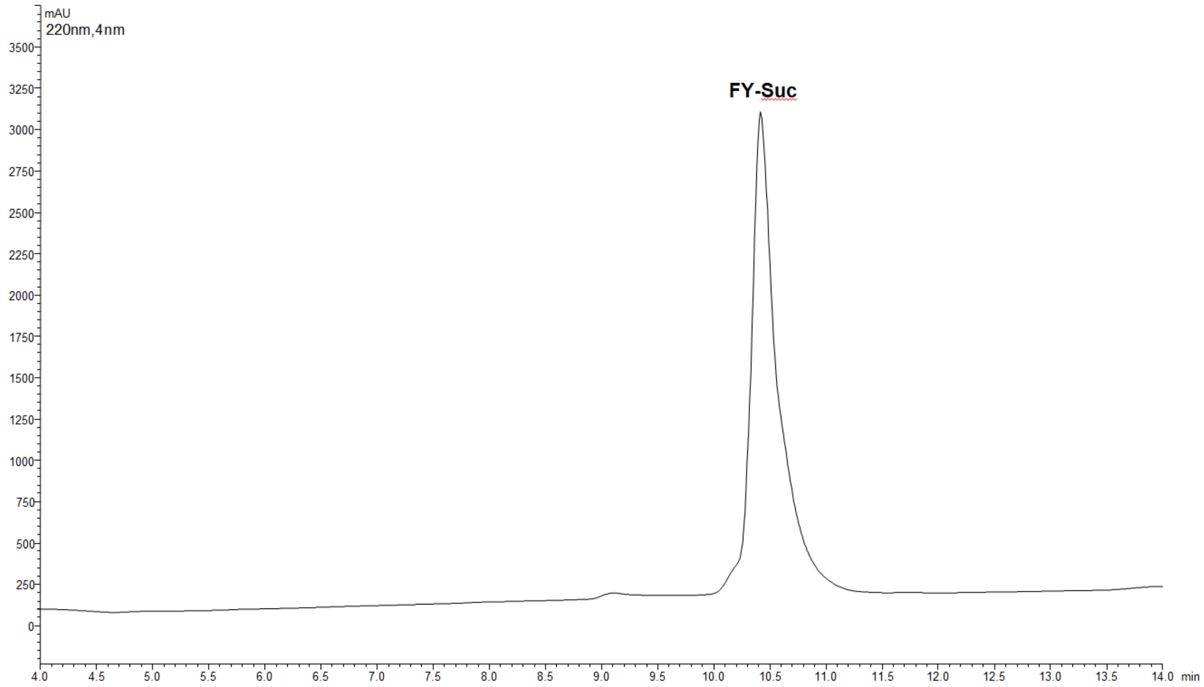

Figure S4. RP-HPLC of **FY-Suc** using analytical column AC2; 0.5 mL/min; gradient: 5–95 % B in 15 min; t_R = 10.5 min.

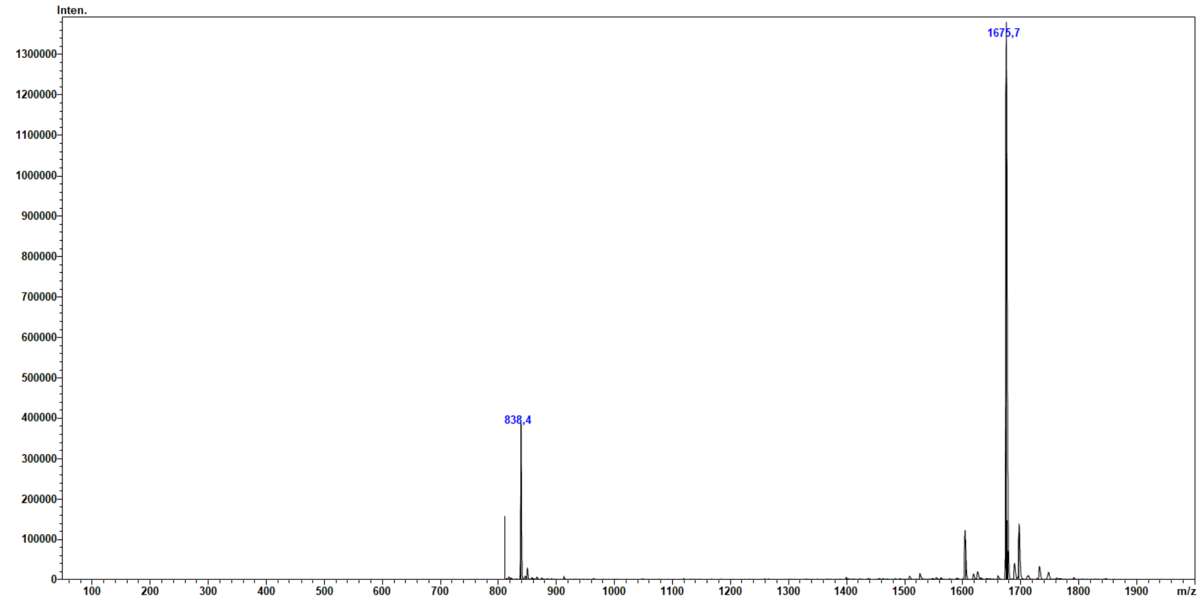

Figure S5. ESI-MS of **FY-Suc-TFP**. m/z corresponds to molecular composition as follows: 1675.7 $[M+H]^{+}$, 838.4 $[M+2H]^{2+}$.

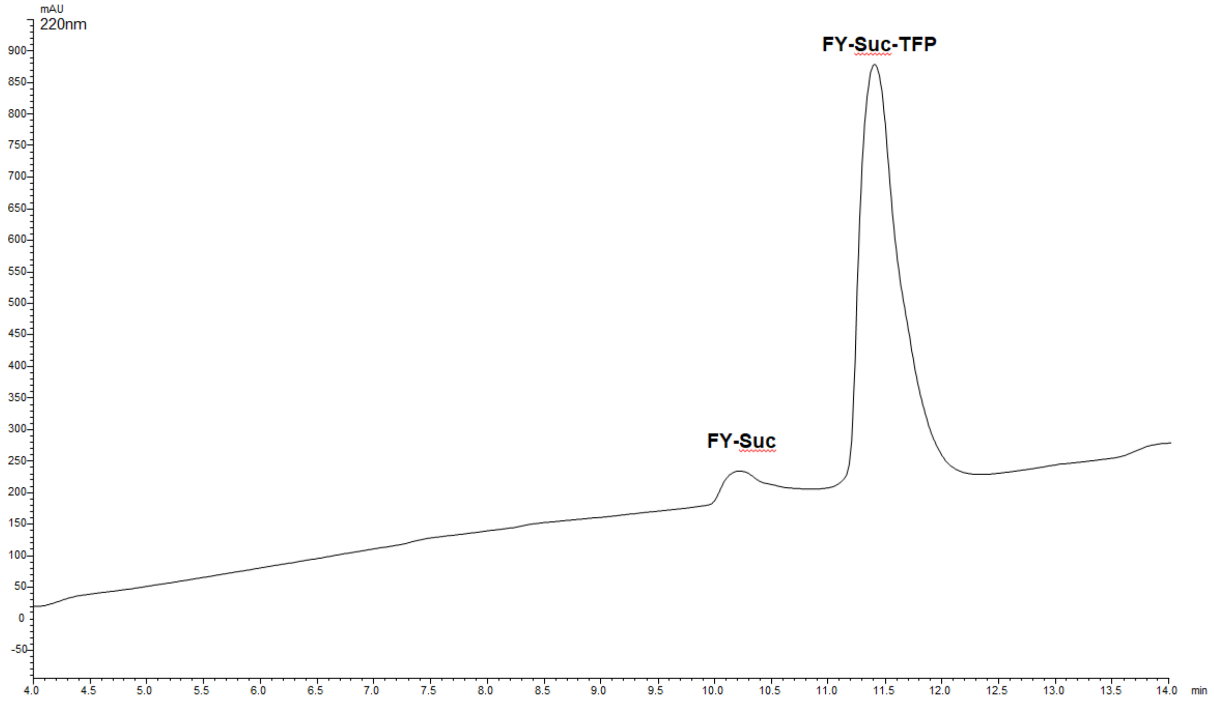

Figure S6. RP-HPLC of **FY-Suc-TFP** using analytical column AC2; 0.5 mL/min; gradient: 10–90 % B in 15 min; t_R = 11.5 min.

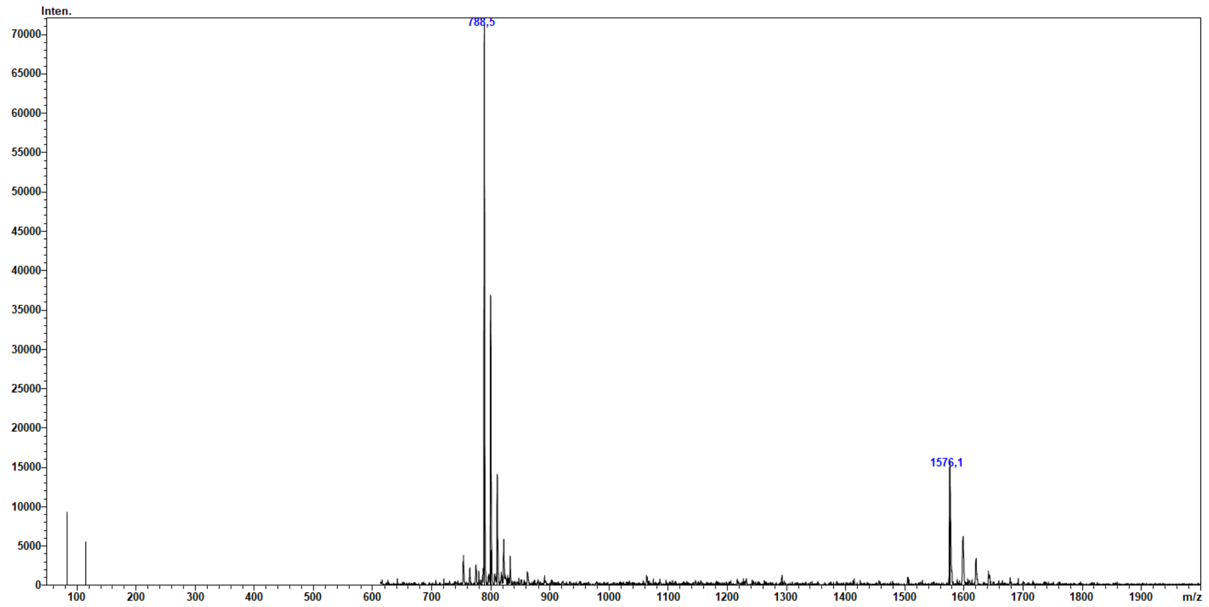

Figure S7. ESI-MS of **GFK-FY**. m/z corresponds to molecular composition as follows: 1576.1 $[M+H^{+}]^{+}$, 788.5 $[M+2H^{+}]^{2+}$.

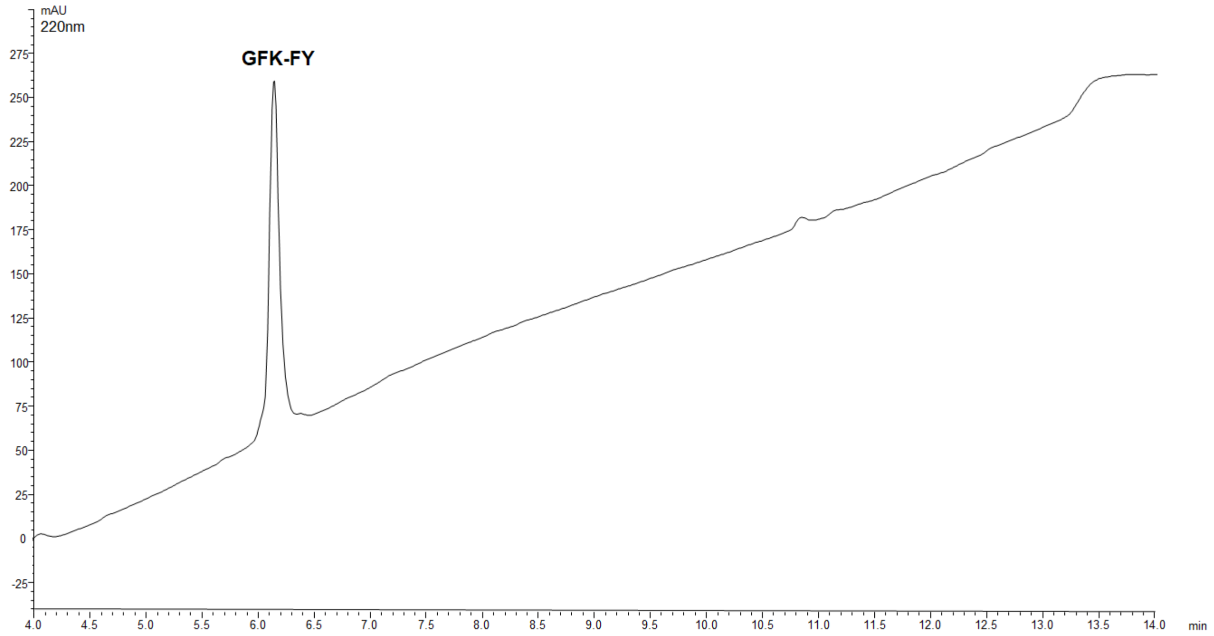

Figure S8. RP-HPLC of **GFK-FY** using analytical column AC2; 0.5 mL/min; gradient: 10–90 % B in 15 min; t_R = 6.2 min.

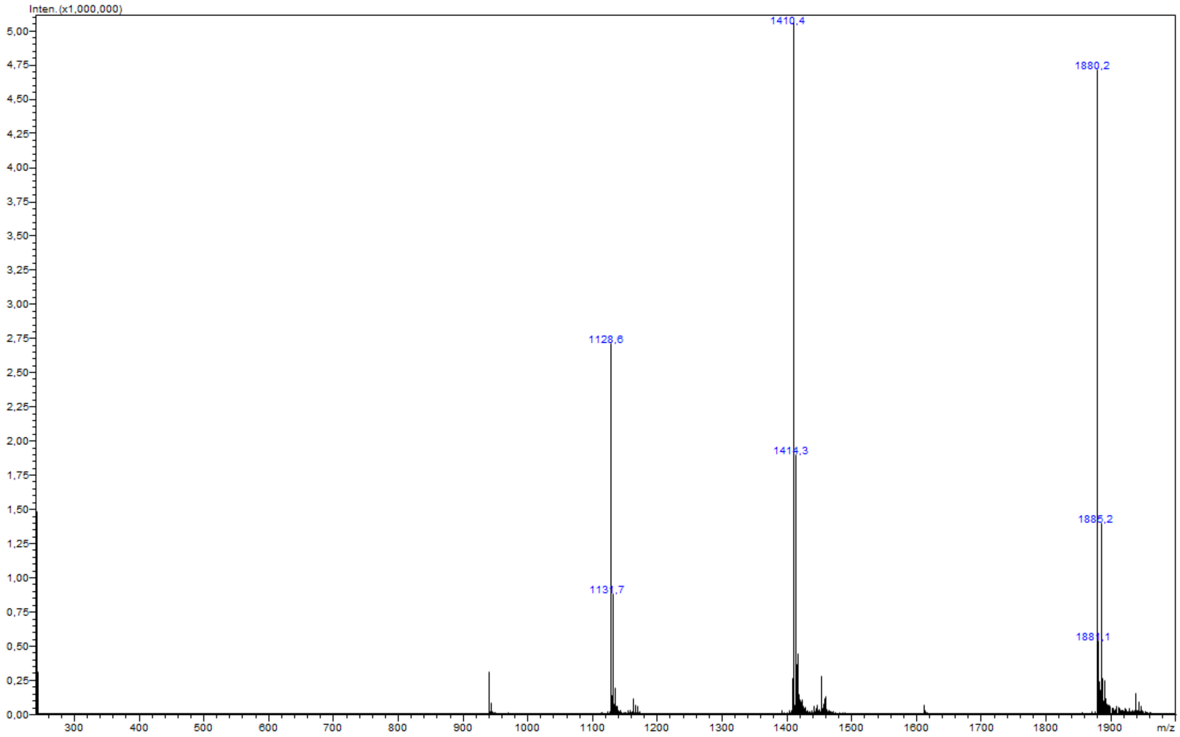

Figure S9. ESI-MS of **Y0**. m/z corresponds to molecular composition as follows: 1880.2 $[M+K^{+}+2H^{+}]^{3+}$, 1410.4 $[M+K^{+}+3H^{+}]^{4+}$, 1128.6 $[M+K^{+}+4H^{+}]^{5+}$.

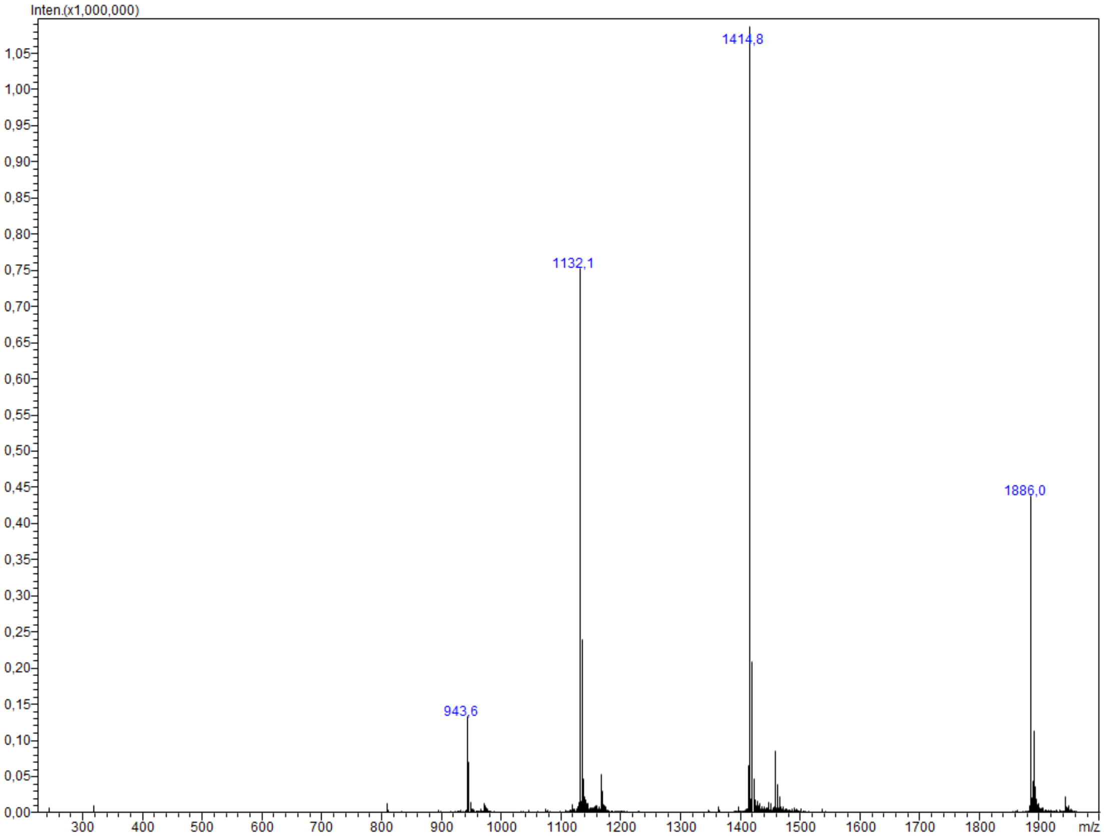

Figure S10. ESI-MS of **Y3**. m/z corresponds to molecular composition as follows: 1886.0 $[M+3H^{+}]^{3+}$, 1414.8 $[M+4H^{+}]^{4+}$, 1132.1 $[M+5H^{+}]^{5+}$, 943.6 $[M+6H^{+}]^{6+}$.

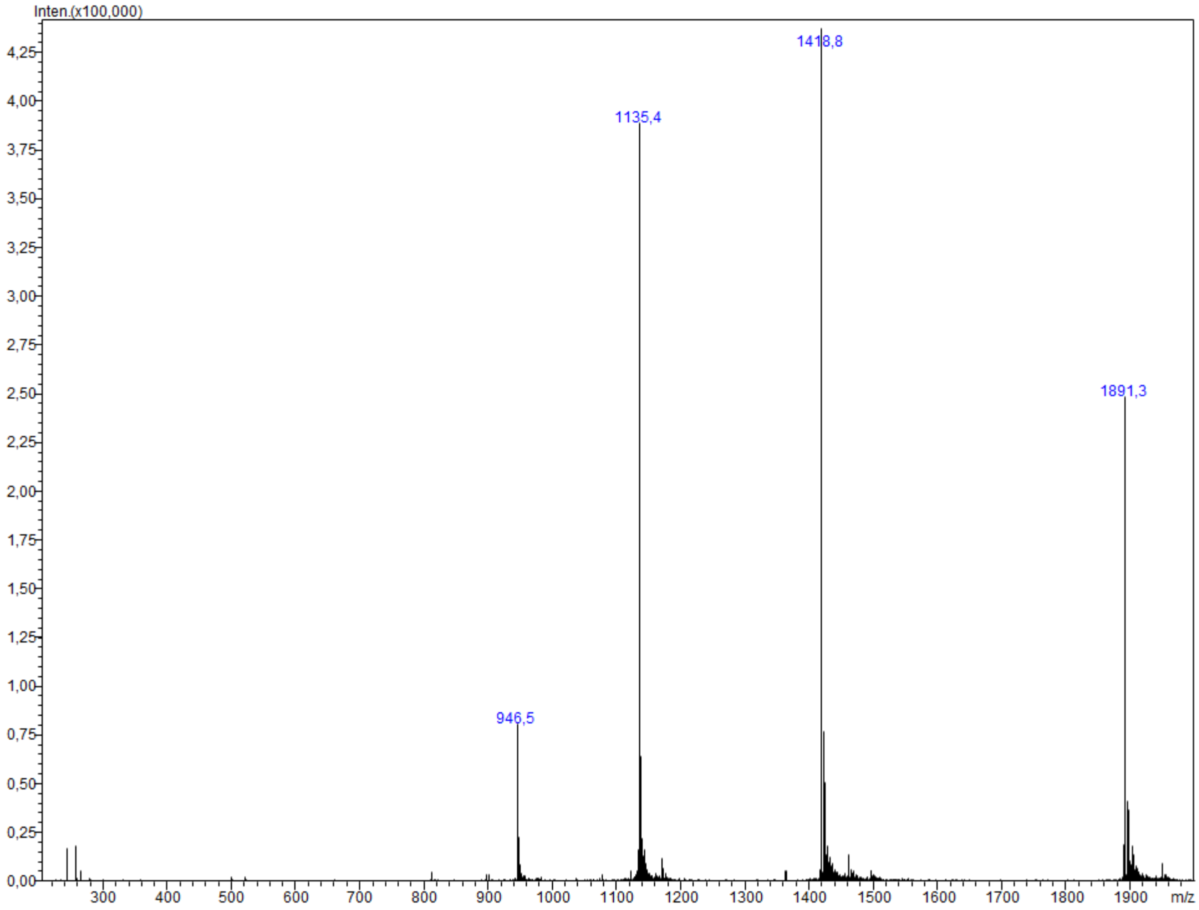

Figure S11. ESI-MS of **Y4**. m/z corresponds to molecular composition as follows: 1891.3 $[M+3H^+]^{3+}$, 1418.9 $[M+4H^+]^{4+}$, 1135.1 $[M+5H^+]^{5+}$, 946.5 $[M+6H^+]^{6+}$.

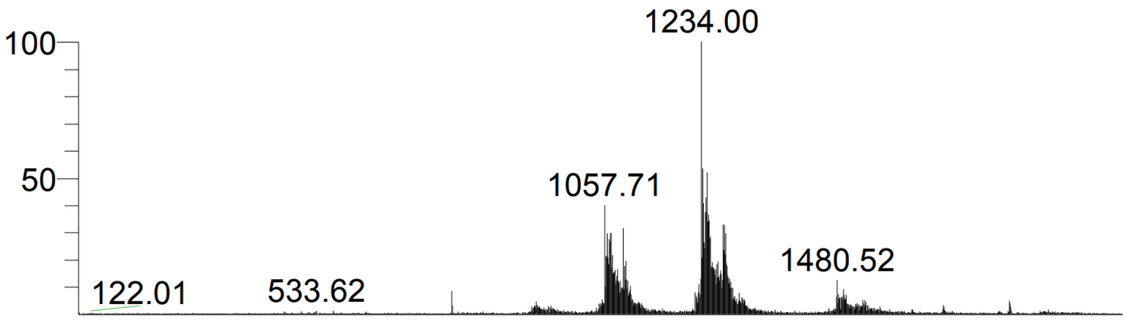

Figure S12. ESI-MS of **GFK-Y4**. *m/z* corresponds to molecular composition as follows: 1480.52 $[M+5H^+]^{5+}$, 1234.00 $[M+6H^+]^{6+}$, 1057.71 $[M+7H^+]^{7+}$.

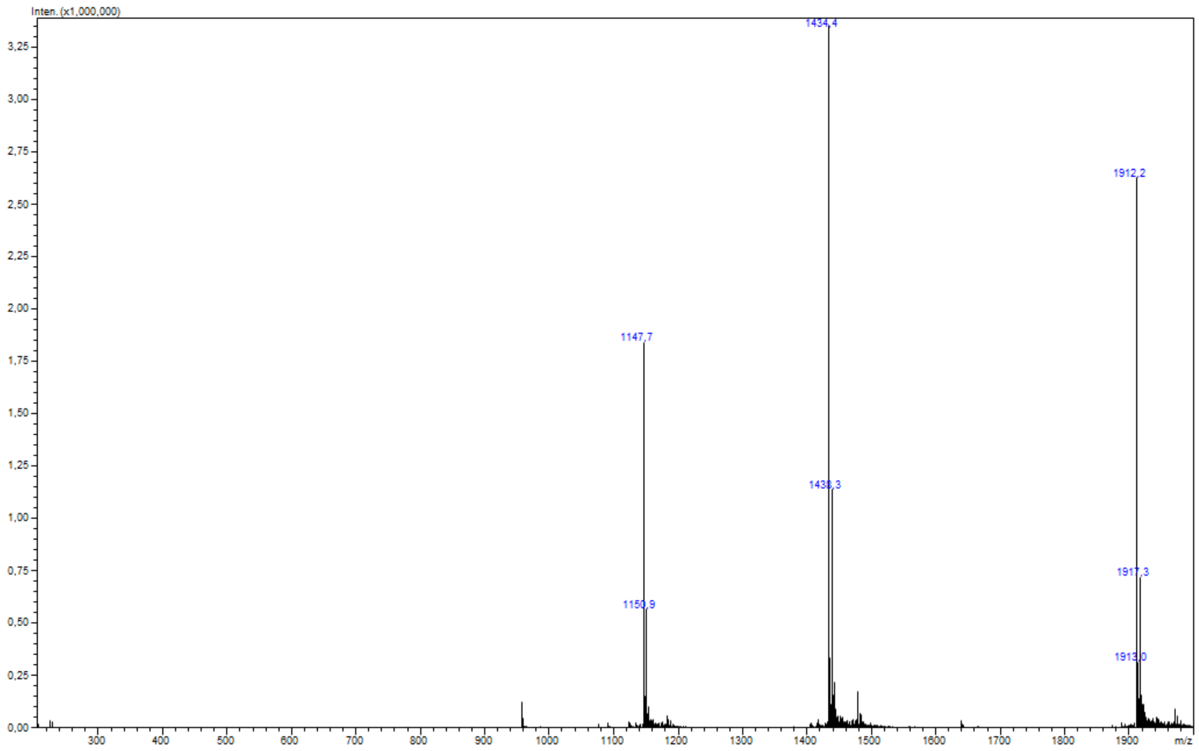

Figure S13. ESI-MS of **Y8**. m/z corresponds to molecular composition as follows: 1912.2 $[M+3H]^{3+}$, 1434.4 $[M+4H]^{4+}$, 1147.7 $[M+5H]^{5+}$.

2. Radio-HPLC

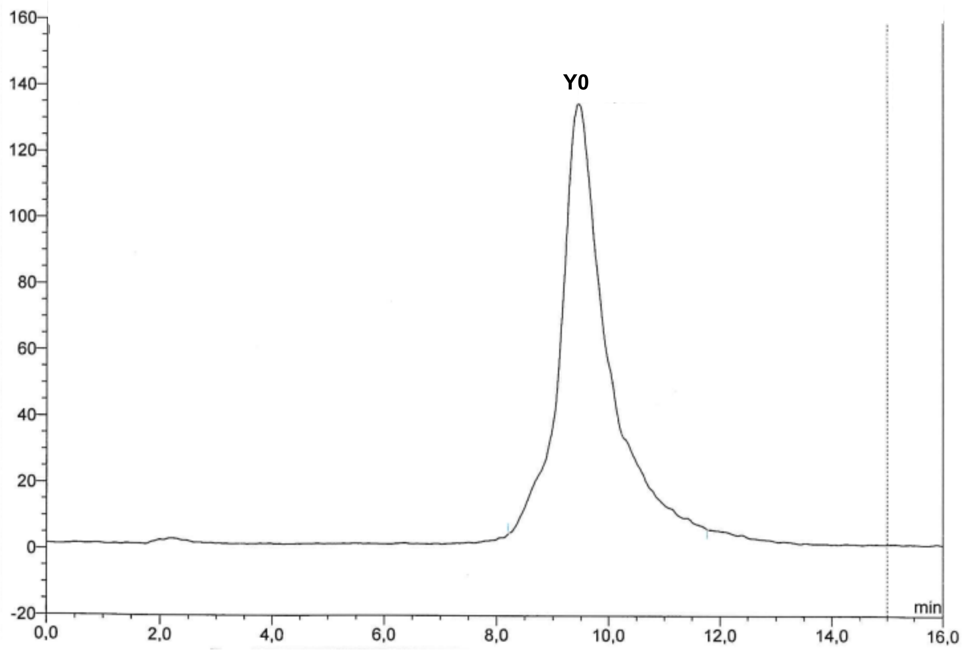

Figure S14. Radio-RP-HPLC of ^{177}Lu-**Y0** performed using analytical column AC1; 1 mL/min; gradient: 0–99 % B in 15 min; t_R = 9.5 min. Radiochemical purity: 98%.

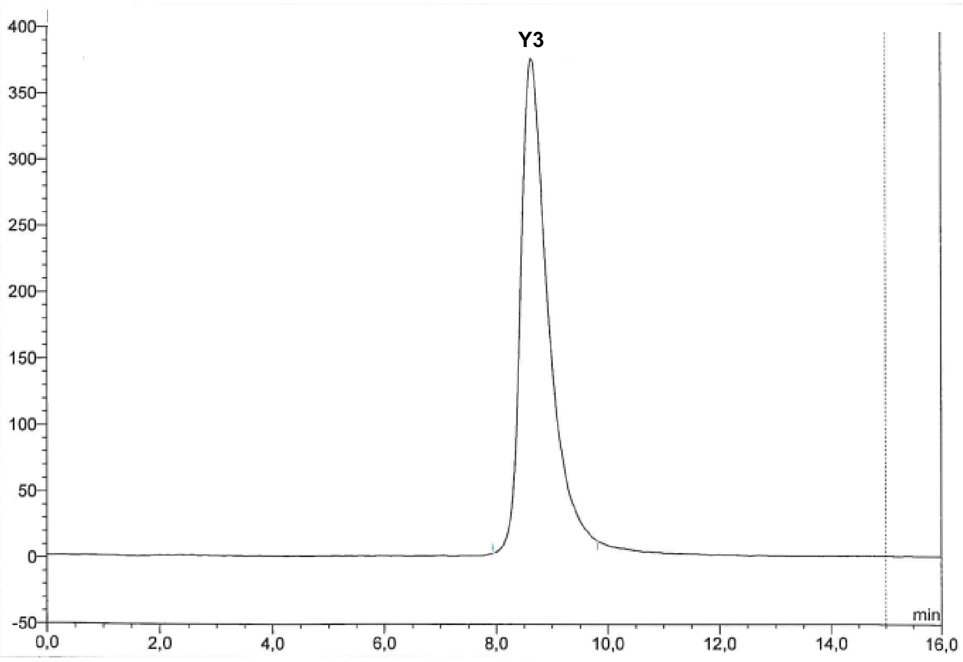

Figure S15. Radio-RP-HPLC of ^{177}Lu-**Y3** performed using analytical column AC1; 1 mL/min; gradient: 0–99 % B in 15 min; t_R = 8.6 min. Radiochemical purity = 100%.

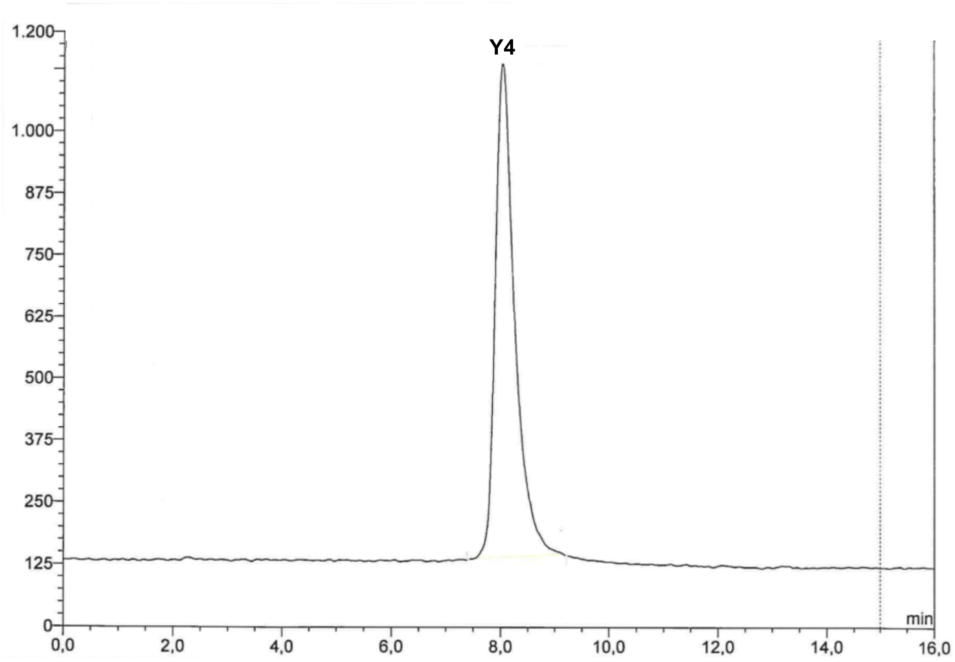

Figure S16. Radio-RP-HPLC of ^{177}Lu-**Y4** performed using analytical column AC1; 1 mL/min; gradient: 0–99 % B in 15 mins; t_R = 8 min. Radiochemical purity = 100%.

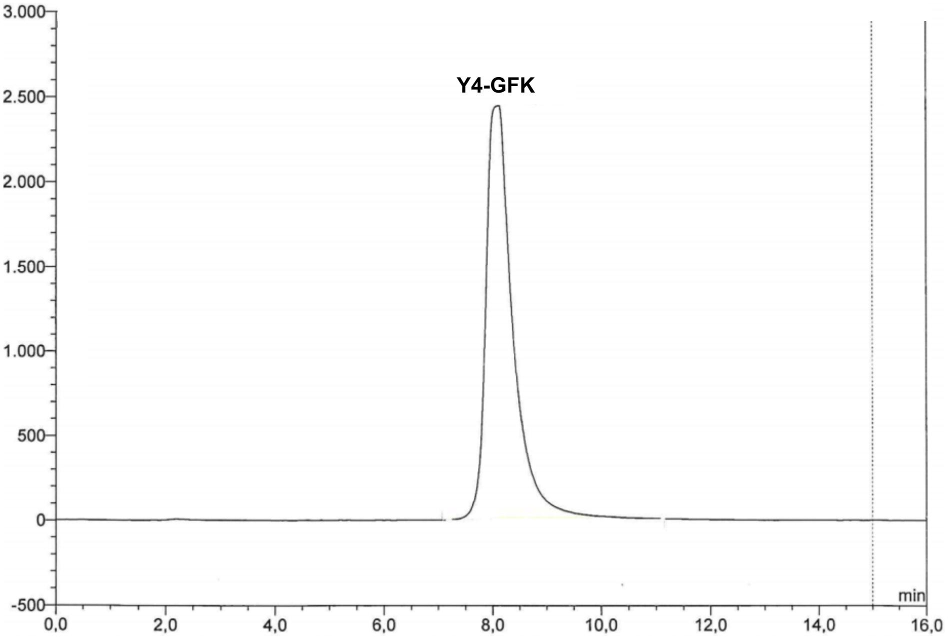

Figure S17. Radio-RP-HPLC of ^{177}Lu-**GFK-Y4** performed using analytical column AC1; 1 mL/min; gradient: 0–99 % B 15 min;. t_R = 8.1 min. Radiochemical purity = 100%.

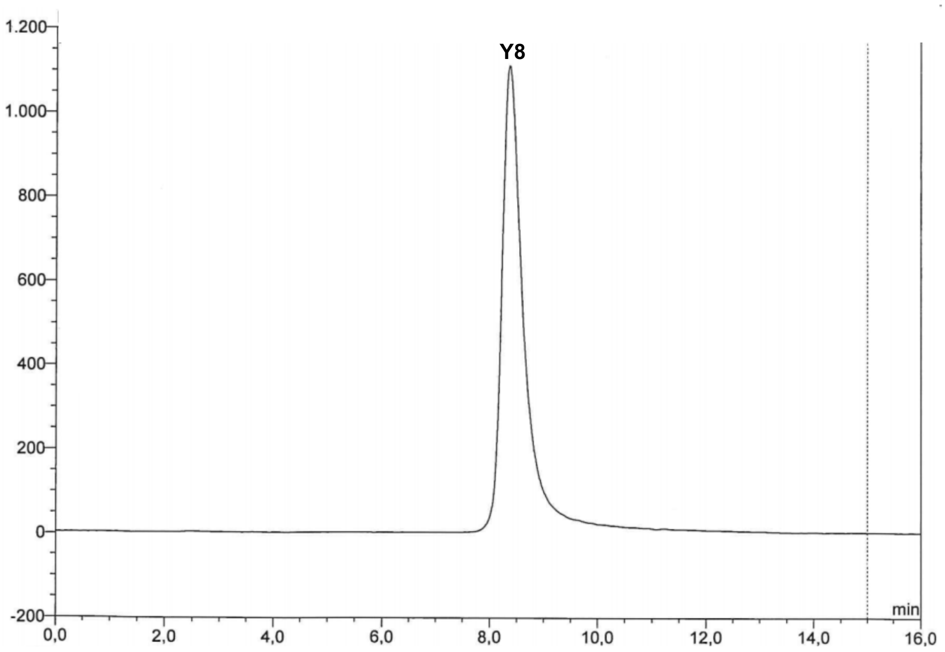

Figure S18. Radio-RP-HPLC of ^{177}Lu-**Y8** performed using analytical column AC1; 1 mL/min; gradient: 0–99 % B in 15 min; t_R = 8.4 min. Radiochemical purity = 100%.

3. Radio-TLC

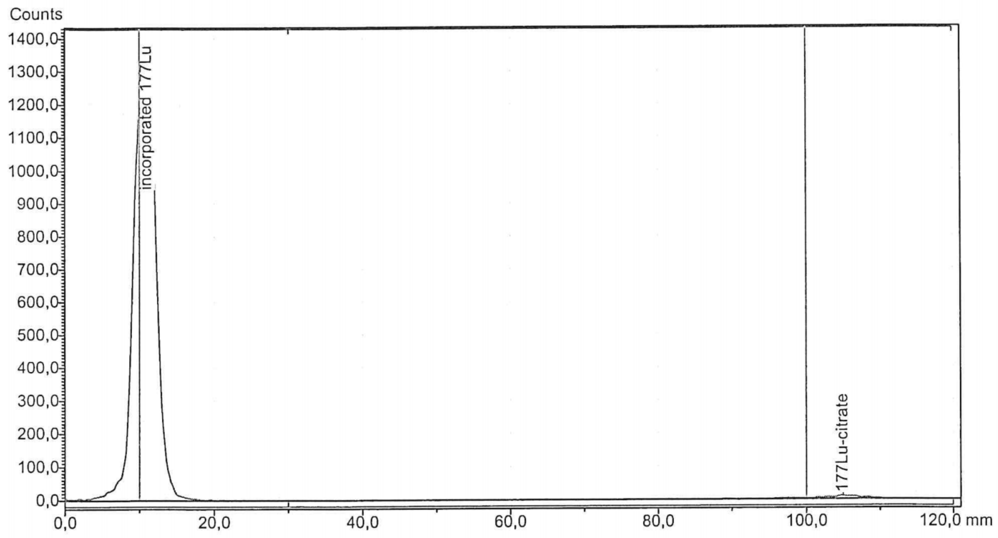

Regions: TLC

Name	Start (mm)	End (mm)	Retention (RF)	Height (Counts)	Area (Counts)	%ROI (%)	%Total (%)
incorporated 177Lu	0,0	30,0	0,0	1379,0	23718,0	98,60	98,60
177Lu-citrate	30,0	120,0	1,1	14,0	337,0	1,40	1,40
2 Peaks					24055,0	100,00	100,00

Figure S19. Radio-TLC for ^{177}Lu-**Y0**; mobile phase: 0.1 M trisodium citrate.

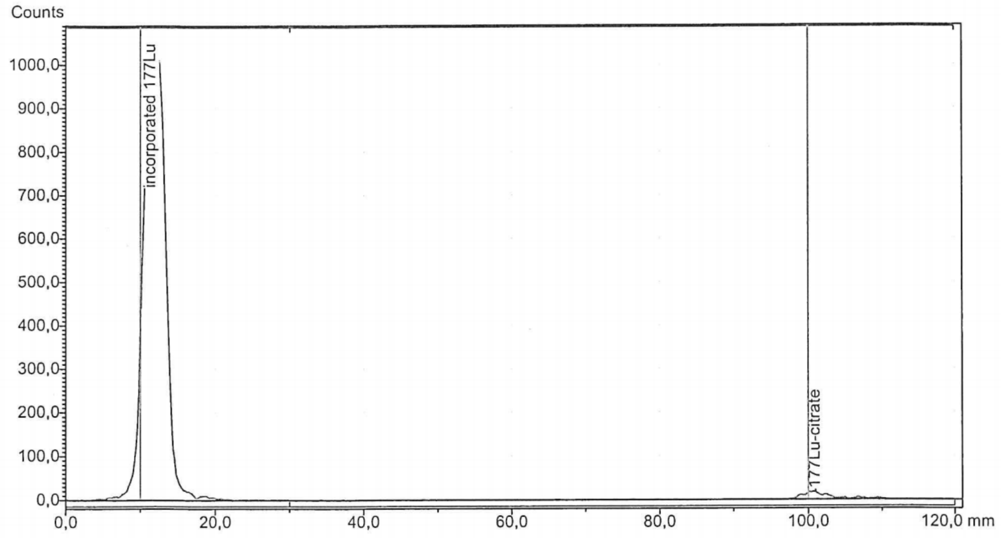

Regions: TLC

Name	Start (mm)	End (mm)	Retention (RF)	Height (Counts)	Area (Counts)	%ROI (%)	%Total (%)
incorporated 177Lu	0,0	30,0	0,0	1049,0	18362,0	97,72	97,72
177Lu-citrate	30,0	120,0	1,0	20,0	428,0	2,28	2,28
2 Peaks					18790,0	100,00	100,00

Figure S20. Radio-TLC for ^{177}Lu-**Y3**; mobile phase: 0.1 M trisodium citrate.

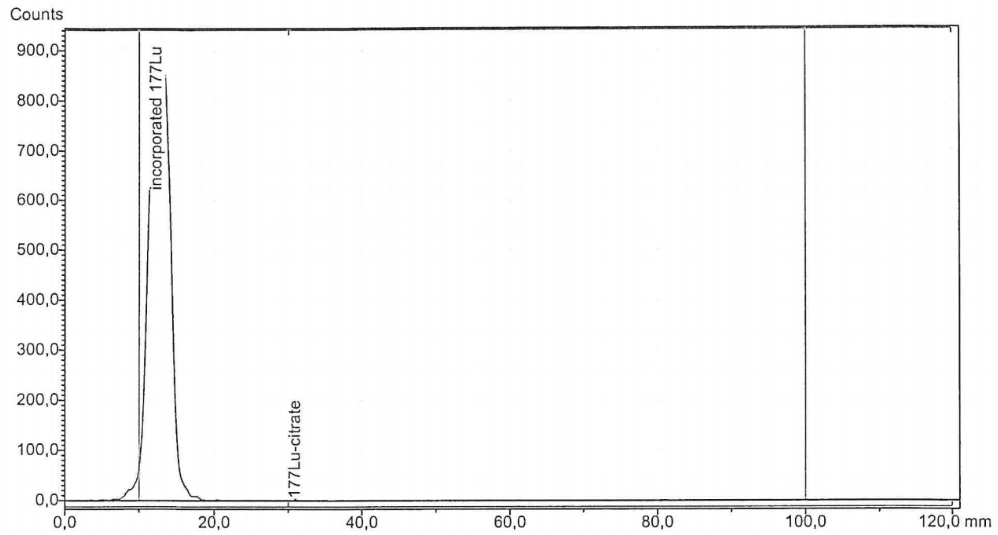

Regions: TLC

Name	Start (mm)	End (mm)	Retention (RF)	Height (Counts)	Area (Counts)	%ROI (%)	%Total (%)
incorporated 177Lu	0,0	30,0	0,0	908,0	15340,0	99,92	99,92
177Lu-citrate	30,0	120,0	0,2	3,0	12,0	0,08	0,08
2 Peaks					15352,0	100,00	100,00

Figure S21. Radio-TLC for ^{177}Lu-**Y4**; mobile phase: 0.1 M trisodium citrate.

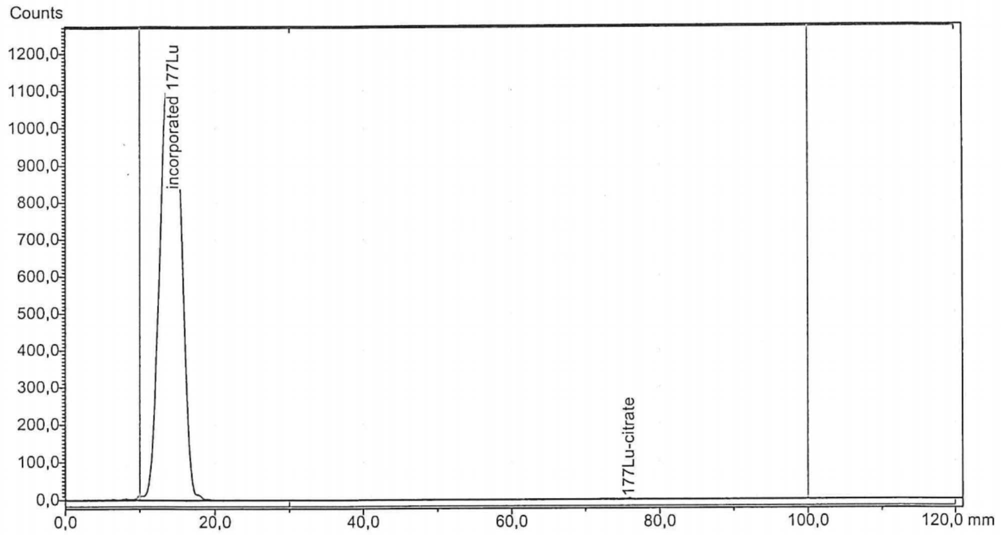

Regions: TLC

Name	Start (mm)	End (mm)	Retention (RF)	Height (Counts)	Area (Counts)	%ROI (%)	%Total (%)
incorporated 177Lu	0,0	30,0	0,1	1225,0	19340,0	99,78	99,78
177Lu-citrate	30,0	120,0	0,7	4,0	43,0	0,22	0,22
2 Peaks					19383,0	100,00	100,00

Figure S22. Radio-TLC for ^{177}Lu-**GFK-Y4**; mobile phase: 0.1 M trisodium citrate.

Chromatogram: TLC

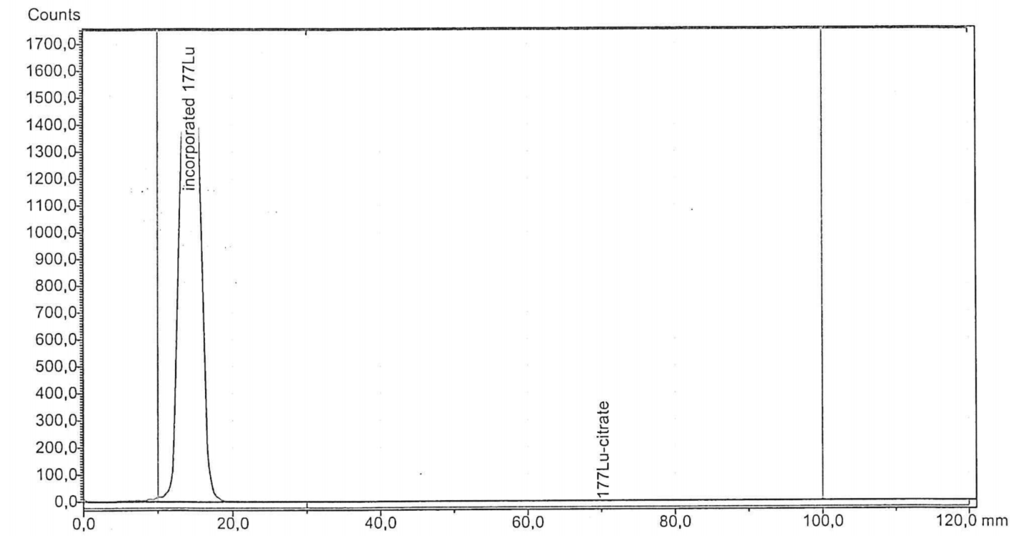

Regions: TLC

Name	Start (mm)	End (mm)	Retention (RF)	Height (Counts)	Area (Counts)	%ROI (%)	%Total (%)
incorporated 177Lu	0,0	30,0	0,0	1690,0	27045,0	99,95	99,95
177Lu-citrate	30,0	120,0	0,7	1,0	14,0	0,05	0,05
2 Peaks					27059,0	100,00	100,00

Figure S23. Radio-TLC for ^{177}Lu-**Y8**; mobile phase: 0.1 M trisodium citrate.

4. Biodistribution data

Table S1: Biodistribution data for ^{177}Lu-**Y0** (*n* = 4; 90 ± 24 pmol), in H2009 bearing SCID mice. Data are given as averages ± standard deviation. %IA/g = percent injected activity per gram tissue.

^{177}Lu-**Y0**	**90 min** p.i.	
Organ/Tissue	%IA/g	tumor/organ ratio
Blood	1.20 ± 0.23	2.7 ± 0.7
Heart (myocard)	1.13 ± 0.62	3.0 ± 0.5
Lung	3.59 ± 1.01	0.9 ± 0.1
Liver	5.03 ± 1.98	0.7 ± 0.2
Spleen	2.22 ± 1.11	1.6 ± 0.4
Pancreas	0.49 ± 0.13	6.7 ± 1.7
Stomach (empty)	4.15 ± 1.03	0.8 ± 0.2
Small intestine (empty)	1.67 ± 0.41	2.0 ± 0.6
Large intestine (empty)	3.00 ± 0.32	1.1 ± 0.4
Kidneys	208 ± 65	0.0 ± 0.0
Adrenals	1.36 ± 0.79	2.9 ± 1.3
Muscle	0.53 ± 0.13	6.0 ± 1.1
Bone	1.45 ± 0.57	2.4 0.7
Tumor H2009	3.23 ± 1.17	

Table S2: Biodistribution data for ^{177}Lu-**Y0** (*n* =5 ; 92 ± 11 pmol), in H2009 bearing SCID mice. Data are given as averages ± standard deviation. %IA/g = percent injected activity per gram tissue.

^{177}Lu-**Y0**	**3 d** p.i.					
Organ/Tissue	%IA/g			tumor/organ ratio		
Blood	0.02	±	0.00	97.5	±	10.4
Heart (myocard)	0.43	±	0.07	4.3	±	0.7
Lung	1.22	±	0.17	1.5	±	0.1
Liver	2.61	±	0.42	0.7	±	0.1
Spleen	1.58	±	0.26	1.1	±	0.2
Pancreas	0.31	±	0.09	6.3	±	1.9
Stomach (empty)	2.63	±	0.68	0.8	±	0.3
Small intestine (empty)	0.82	±	0.25	2.2	±	0.6
Large intestine (empty)	1.19	±	0.53	2.1	±	1.5
Kidneys	243	±	56	0.0	±	0.0
Adrenals	0.82	±	0.06	2.1	±	0.1
Muscle	0.29	±	0.11	8.2	±	6.4
Bone	0.42	±	0.14	5.0		2.4
Tumor H2009	1.72	±	0.07			

Table S3: Biodistribution data for ^{177}Lu-**Y3** (*n* = 3; 139 ± 59 pmol), in H2009 bearing SCID mice. Data are given as averages ± standard deviation. %IA/g = percent injected activity per gram tissue.

^{177}Lu-**Y3**	**90 min** p.i.					
Organ/Tissue	%IA/g			tumor/organ ratio		
Blood	1.11	±	0.14	3.4	±	0.4
Heart (myocard)	0.65	±	0.12	5.8	±	0.7
Lung	4.91	±	0.51	0.8	±	0.1
Liver	7.59	±	3.21	0.6	±	0.3
Spleen	8.50	±	2.27	0.5	±	0.1
Pancreas	0.36	±	0.05	10.3	±	1.2
Stomach (empty)	7.42	±	0.32	0.5	±	0.0
Small intestine (empty)	2.49	±	0.46	1.5	±	0.2
Large intestine (empty)	3.43	±	0.68	1.1	±	0.2
Kidneys	141	±	30	0.0	±	0.0
Adrenals	1.04	±	0.44	3.9	±	1.2
Muscle	0.57	±	0.10	6.6	±	0.9
Bone	1.51	±	0.14	2.5		0.1
Tumor H2009	3.70	±	0.16			

Table S4: Biodistribution data for ^{177}Lu-**Y3** (*n* =5 ; 42 ± 6 pmol), in H2009 bearing SCID mice. Data are given as averages ± standard deviation. %IA/g = percent injected activity per gram tissue.

^{177}Lu-**Y3**	**3 d** p.i.					
Organ/Tissue	%IA/g			tumor/organ ratio		
Blood	0.02	±	0.00	128.6	±	22.2
Heart (myocard)	0.22	±	0.04	9.4	±	2.6
Lung	1.22	±	0.16	1.7	±	0.4
Liver	1.17	±	0.33	1.9	±	0.6
Spleen	1.36	±	0.54	1.7	±	0.6
Pancreas	0.25	±	0.17	10.6	±	4.8
Stomach (empty)	4.76	±	1.37	0.5	±	0.2
Small intestine (empty)	0.37	±	0.18	6.6	±	3.2
Large intestine (empty)	1.34	±	0.72	2.3	±	1.9
Kidneys	250	±	33	0.0	±	0.0
Adrenals	0.27	±	0.11	8.4	±	3.8
Muscle	0.47	±	0.23	5.3	±	2.6
Bone	0.35	±	0.17	6.6		2.5
Tumor H2009	2.03	±	0.29			

Table S5: Biodistribution data for ^{177}Lu-**Y4** (*n* = 4; 43 ± 7 pmol), in H2009 bearing SCID mice. Data are given as averages ± standard deviation. %IA/g = percent injected activity per gram tissue.

^{177}Lu-**Y4**	**90 min** p.i.					
Organ/Tissue	%IA/g			tumor/organ ratio		
Blood	1.33	±	0.10	5.2	±	0.6
Heart (myocard)	0.74	±	0.19	9.6	±	1.6
Lung	6.74	±	0.71	1.0	±	0.1
Liver	1.06	±	0.13	6.6	±	1.3
Spleen	4.71	±	1.04	1.5	±	0.4
Pancreas	0.37	±	0.02	18.6	±	2.2
Stomach (empty)	10.65	±	3.09	0.7	±	0.2
Small intestine (empty)	3.05	±	1.08	2.6	±	1.5
Large intestine (empty)	6.99	±	0.20	1.0	±	0.1
Kidneys	219	±	17	0.0	±	0.0
Adrenals	0.52	±	0.20	13.9	±	3.4
Muscle	1.00	±	0.44	8.2	±	4.1
Bone	1.84	±	0.20	3.8		0.4
Tumor H2009	6.91	±	0.73			

Table S6: Biodistribution data for ^{177}Lu-**Y4** (*n* = 5; 64 ± 6 pmol), in H2009 bearing SCID mice. Data are given as averages ± standard deviation. %IA/g = percent injected activity per gram tissue.

^{177}Lu-**Y4**	**3 d** p.i.					
Organ/Tissue	%IA/g			tumor/organ ratio		
Blood	0.02	±	0.01	108.2	±	40.0
Heart (myocard)	0.27	±	0.08	8.2	±	2.3
Lung	1.21	±	0.24	1.7	±	0.3
Liver	0.88	±	0.33	2.6	±	0.8
Spleen	1.31	±	0.33	1.7	±	0.4
Pancreas	0.19	±	0.04	11.3	±	1.3
Stomach (empty)	3.88	±	0.80	0.6	±	0.1
Small intestine (empty)	0.26	±	0.06	8.2	±	1.7
Large intestine (empty)	1.33	±	0.26	1.6	±	0.3
Kidneys	203	±	18	0.0	±	0.0
Adrenals	0.90	±	0.66	4.1	±	3.3
Muscle	0.28	±	0.12	9.1	±	5.4
Bone	0.55	±	0.07	3.8		0.8
Tumor H2009	2.07	±	0.16			

Table S7: Biodistribution data for ^{177}Lu-**GKF-Y4** (*n* = 4; 118 ± 40 pmol), in H2009 bearing SCID mice. Data are given as averages ± standard deviation. %IA/g = percent injected activity per gram tissue.

^{177}Lu-**GFK-Y4**	**90 min** p.i.					
Organ/Tissue	%IA/g			tumor/organ ratio		
Blood	0.44	±	0.09	4.3	±	2.0
Heart (myocard)	0.32	±	0.01	5.5	±	1.6
Lung	2.88	±	0.37	0.6	±	0.2
Liver	1.16	±	0.56	1.7	±	0.6
Spleen	0.89	±	0.22	2.1	±	0.9
Pancreas	0.14	±	0.01	12.2	±	3.7
Stomach (empty)	3.29	±	0.53	0.6	±	0.2
Small intestine (empty)	0.73	±	0.14	2.5	±	1.0
Large intestine (empty)	1.76	±	0.55	1.1	±	0.7
Kidneys	274	±	75	0.0	±	0.0
Adrenals	0.95	±	0.66	2.2	±	0.9
Muscle	0.26	±	0.06	7.2	±	3.0
Bone	0.49	±	0.03	3.6		1.1
Tumor H2009	1.74	±	0.48			

Table S8: Biodistribution data for ^{177}Lu-**GFK-Y4** (*n* = 5; 70 ± 39 pmol), in H2009 bearing SCID mice. Data are given as averages ± standard deviation. %IA/g = percent injected activity per gram tissue.

^{177}Lu-**GFK-Y4**	**3 d** p.i.					
Organ/Tissue	%IA/g			tumor/organ ratio		
Blood	0.02	±	0.01	56.1	±	25.7
Heart (myocard)	0.12	±	0.03	6.9	±	2.1
Lung	0.50	±	0.26	1.8	±	0.7
Liver	0.48	±	0.22	2.0	±	1.1
Spleen	0.86	±	0.37	1.0	±	0.4
Pancreas	0.08	±	0.02	9.9	±	4.1
Stomach (empty)	2.42	±	0.83	0.4	±	0.1
Small intestine (empty)	0.15	±	0.04	5.6	±	1.9
Large intestine (empty)	0.78	±	0.42	1.4	±	1.0
Kidneys	169	±	37	0.0	±	0.0
Adrenals	0.25	±	0.10	3.7	±	0.9
Muscle	0.13	±	0.06	6.9	±	3.9
Bone	0.25	±	0.06	3.2		1.2
Tumor H2009	0.77	±	0.19			

Table S9: Biodistribution data (min p.i.) for ^{177}Lu-**Y8** without (*n* = 4; 124 ± 24 pmol) and with (n = 2) addition of 50 nmol of non-labeled compound, in H2009 bearing SCID mice. Data are given as averages ± standard deviation. %IA/g = percent injected activity per gram tissue.

^{177}Lu-**Y8**	**90 min** p.i.						+ 50 nmol cold		
Organ/Tissue	%IA/g			tumor/organ ratio			10 min prior to activity		
Blood	2.54	±	0.56	2.4	±	0.5	1.06	±	0.31
Heart (myocard)	1.01	±	0.19	5.9	±	1.3	0.64	±	0.14
Lung	5.63	±	0.44	1.0	±	0.1	3.39	±	0.70
Liver	1.16	±	0.15	5.1	±	1.0	0.70	±	0.06
Spleen	8.41	±	1.93	0.7	±	0.3	4.99	±	0.34
Pancreas	0.42	±	0.08	14.3	±	3.6	0.19	±	0.01
Stomach (empty)	8.26	±	0.91	0.7	±	0.2	1.19	±	0.30
Small intestine (empty)	3.00	±	0.61	2.1	±	0.8	0.62	±	0.09
Large intestine (empty)	3.66	±	1.06	1.8	±	0.8	0.89	±	0.14
Kidneys	166	±	26	0.0	±	0.0	178	±	7
Adrenals	0.71	±	0.20	8.9	±	3.7	0.53	±	0.21
Muscle	0.87	±	0.35	7.4	±	2.7	0.20	±	0.08
Bone	3.24		0.20	1.8		0.4	0.76		0.36
Tumor H2009	5.89	±	1.09				1.71	±	0.41

Table S10: Biodistribution data for ^{177}Lu-**Y8** (*n* = 5; 154 ± 14 pmol), in H2009 bearing SCID mice. Data are given as averages ± standard deviation. %IA/g = percent injected activity per gram tissue.

^{177}Lu-**Y8**	**24 h** p.i.					
Organ/Tissue	%IA/g			tumor/organ ratio		
Blood	0.04	±	0.01	59.6	±	9.9
Heart (myocard)	0.16	±	0.03	15.2	±	3.6
Lung	1.20	±	0.19	2.1	±	0.7
Liver	0.98	±	0.40	2.7	±	1.2
Spleen	1.89	±	0.77	1.4	±	0.6
Pancreas	0.14	±	0.02	18.4	±	6.0
Stomach (empty)	3.84	±	1.33	0.7	±	0.4
Small intestine (empty)	1.03	±	0.27	2.5	±	1.0
Large intestine (empty)	1.46	±	0.55	1.8	±	0.7
Kidneys	235	±	49	0.0	±	0.0
Adrenals	0.25	±	0.08	10.2	±	2.8
Muscle	0.36	±	0.34	9.5	±	4.5
Bone	0.68	±	0.08	3.6		1.0
Tumor H2009	2.42	±	0.58			

Table S11: Biodistribution data for ^{177}Lu-**Y8** (*n* = 8; 199 ± 84 pmol), in H2009 bearing SCID mice. Data are given as averages ± standard deviation. %IA/g = percent injected activity per gram tissue.

^{177}Lu-**Y8**	**3 d** p.i.					
Organ/Tissue	%IA/g			tumor/organ ratio		
Blood	0.03	±	0.01	67.0	±	27.8
Heart (myocard)	0.13	±	0.02	12.2	±	4.0
Lung	0.73	±	0.13	2.2	±	0.8
Liver	0.70	±	0.14	2.4	±	1.0
Spleen	2.01	±	0.71	1.0	±	0.6
Pancreas	0.12	±	0.04	14.9	±	7.1
Stomach (empty)	2.65	±	1.11	0.8	±	0.5
Small intestine (empty)	0.16	±	0.05	11.4	±	5.7
Large intestine (empty)	0.48	±	0.17	3.6	±	1.4
Kidneys	237	±	42	0.0	±	0.0
Adrenals	0.22	±	0.04	4.5	±	2.6
Muscle	0.23	±	0.11	8.4	±	4.1
Bone	0.57	±	0.21	3.4		1.8
Tumor H2009	1.67	±	0.56			
